# Cortical interactions during the resolution of information processing demands in autism spectrum disorders

**DOI:** 10.1002/brb3.596

**Published:** 2016-12-24

**Authors:** Kristina Denisova, Guihu Zhao, Zhishun Wang, Suzanne Goh, Yuankai Huo, Bradley S. Peterson

**Affiliations:** ^1^ Department of PsychiatryCenter for Developmental NeuropsychiatryColumbia University College of Physicians and SurgeonsNew YorkNYUSA; ^2^ Division of Developmental NeuroscienceNew York State Psychiatric InstituteNew YorkNYUSA; ^3^ Sackler Institute for Developmental PsychobiologyColumbia University College of Physicians and SurgeonsNew YorkNYUSA; ^4^ School of Information Science and EngineeringCentral South UniversityChangshaChina; ^5^ Children's Hospital Los AngelesKeck School of Medicine of the University of Southern CaliforniaLos AngelesCAUSA

**Keywords:** autism spectrum disorders, functional connectivity, functional magnetic resonance imaging, psychophysiological interaction, uncertainty

## Abstract

**Introduction:**

Our flexible and adaptive interactions with the environment are guided by our individual representation of the physical world, estimated through sensation and evaluation of available information against prior knowledge. When linking sensory evidence with higher‐level expectations for action, the central nervous system (CNS) in typically developing (TD) individuals relies in part on distributed and interacting cortical regions to communicate neuronal signals flexibly across the brain. Increasing evidence suggests that the balance between levels of signal and noise during information processing may be disrupted in individuals with Autism Spectrum Disorders (ASD).

**Methods:**

Participants with and without ASD performed a visuospatial interference task while undergoing functional Magnetic Resonance Imaging (fMRI). We empirically estimated parameters characterizing participants’ latencies and their subtle fluctuations (noise accumulation) over the 16‐min scan. We modeled hemodynamic activation and used seed‐based analyses of neural coupling to study dysfunction in interference‐specific connectivity in a subset of ASD participants who were nonparametrically matched to TD participants on age, male‐to‐female ratio, and magnitude of movement during the scan.

**Results:**

Stochastic patterns of response fluctuations reveal significantly higher noise‐to‐signal levels and a more random and noisy structure in ASD versus TD participants, and in particular ASD adults who have the greatest clinical autistic deficits. While individuals with ASD show an overall weaker modulation of interference‐specific functional connectivity relative to TD individuals, in particular between the seeds of Anterior Cingulate Cortex (ACC) and Inferior Parietal Sulcus (IPS) and the rest of the brain, we found that in ASD, higher uncertainty during the task is linked to increased interference‐specific coupling between bilateral anterior insula and prefrontal cortex.

**Conclusions:**

Subtle and informative differences in the structure of experiencing information exist between ASD and TD individuals. Our findings reveal in ASD an atypical capacity to apply previously perceived information in a manner optimal for adaptive functioning, plausibly revealing suboptimal message‐passing across the CNS.

## 1 Introduction

The central nervous system samples inherently ambiguous and underdetermined raw sensory inputs and compares them to an internal model constructed from prior knowledge of the environment (Helmholtz, 1925). Humans sense, perceive, evaluate, and act upon the basis of this comparison, with the CNS continuously updating its internal representation of the world (Knill & Pouget, 2004). Physiological limits on the rate of neural spiking and the finite availability of energy (Lennie, 2003) require the parsimonious communication between concurrently active neurons when performing a task, a requirement instantiated in part via cooperative message‐passing across levels of the cortical hierarchy. The interactions of feed‐forward messaging (i.e., signaling deviations from expectations at lower sensory levels and updating conditional expectations in higher levels Rahnev, Lau, & de Lange, 2011) and top‐down signaling (i.e., relaying task‐set expectations and modulating sensory representations) (Kayser, Erickson, Buchsbaum, & D'Esposito, 2010) require synergetic neuronal activity across broadly distributed brain regions (Friston, 2010).

Little is understood about how individuals with Autism Spectrum Disorders (ASD) build and sustain their experience of the world. Previous research in ASD suggests the presence of variable, unreliable, or noisy cortical responses to sensory stimuli (Dinstein et al., 2012) and noise accumulation over time during simple and complex movements (e.g., Torres et al., 2016). Spontaneous, resting‐state functional connectivity MRI (rs‐fcMRI) studies report evidence for atypicalities in both local‐ and global‐scale network processing in ASD (e.g., Ecker et al., 2013; Khan et al., 2013), suggesting the presence of compromised communication among brain regions, although recent work has questioned the underconnectivity hypothesis, with some researchers finding normal resting‐state patterns in individuals with ASD (e.g., Tyszka, Kennedy, Paul, & Adolphs, 2014).

Herein, we manipulated the levels of visuospatial interference during an fMRI scan (Peterson et al., 2002) to test the theory of a temporal binding deficit in autism (Brock, Brown, Boucher, & Rippon, 2002), which posits that reduced temporal synchrony (in‐phase neuronal firing) between remote neuronal populations impairs the integration and coordination of information in ASD. Two hypotheses were considered. First, we asked if individuals with ASD would still show atypical temporal correlation across the brain during the task, when ASD and TD groups are stringently matched on the amount of head movement during the scan. In particular, because information flow along long‐distance fibers that interconnect frontal and parietal cortices supports the synergetic activity mediating top‐down and feed‐forward processing that may be aberrant in autism (Belmonte et al., 2004; Just, Cherkassky, Keller, & Minshew, 2004), we hypothesized that ASD individuals would show aberrant interference‐specific functional coupling between frontal and parietal cortices when responding to a less rather than more automatically processed dimension of a stimulus (i.e., the direction rather than spatial position of an arrow) (Brock et al., 2002). Second, we asked if individuals with ASD can be characterized (using the simple task here) by the differences in their noise‐to‐signal levels in responding to the task. We use fluctuation analysis to estimate and characterize empirically the stochasticity of response patterns during the scan (patterns of subtle fluctuation in reaction times, RTs). Evidence for altered interference‐specific functional coupling across the brain in ASD and statistically suboptimal stochastic patterns in behavior would indicate altered communication of information across the brain, and thereby would provide insight into the interplay between perception, thought and action in ASD.

## 2 Materials and Methods

### 2.1 Participants

Individuals with ASD were recruited through the Developmental Neuropsychiatry Clinic at Columbia University and community awareness events, whereas typically developing (TD) participants were recruited through flyers, online advertisements, and via a telemarketing list of random households eligible for participation. Demographic characteristics are presented in Table 1. We screened potential ASD participants for participation by requiring them to have a previous clinical DSM‐IV (Association, 2000) diagnosis of ASD and to meet a cutoff of >15 on either the Social Communication Questionnaire (SCQ; Rutter, Bailey, & Lord, 2003), Lifetime Version or on the Autism‐spectrum Quotient (AQ) (Baron‐Cohen, Wheelwright, Skinner, Martin, & Clubley, 2001). Potential ASD participants were excluded on the basis of known medical conditions associated with autism (e.g., Fragile‐X syndrome and tuberous sclerosis), premature birth or low birth weight (<36 weeks of gestation and <2000 g at birth), obstetrical complications, or a known seizure disorder. The final, best estimate diagnoses were established by a physician or clinical psychologist with established reliability by administering Module 2 (*n *= 2), 3 (*n *= 20), or 4(*n *= 28) of the Autism Diagnostic Observation Schedule (ADOS‐G; Lord et al., 2000). The revised algorithm was used for all modules (Bastiaansen et al., 2011; Gotham, Risi, Pickles, & Lord, 2007). Diagnoses for younger participants were also established by administering the parent Autism Diagnostic Interview‐Revised (ADI‐R; Lord, Rutter, & Le Couteur, 1994; *n *= 23). Three of the 50 ASD participants who did not meet the screening cutoffs on the SCQ or AQ were admitted to the study on the basis of a previous ADOS‐based diagnosis. The final ASD group comprised individuals with Autistic Disorder (*n *= 17), Asperger's Disorder (*n *= 25), and Pervasive Developmental Disorder Not Otherwise Specified (PDD‐NOS; *n *= 8). Note that according to the most recent version of DSM (DSM5), individuals who previously would have received these separate diagnoses now receive a diagnosis of ASD.

**Table 1:** Demographic characteristics of the main sample

	ASD (*n *= 50)	TD (*n *= 50)	Analysis (df)	*p*
Age (years)	27.10 ± 13.04	26.32 ± 12.30	*t*_(98)_ = 0.31	.76
Sex, M/F	42/9	42/9	*Χ* ^2^(1) = 0	.99
Caucasian	40W/10 non‐W	37W/13 non‐W	*Χ* ^2^(1) = 1.13	.29
Full Scale IQ^a^	110.74 ± 18.24	116.41 ± 11.39	*t*_(94)_ = −1.83	.07
SRS autistic mannerisms^b^	15.85 ± 6.56	2.08 ± 2.28	*t*_(70)_ = 11.17	<.01
SRS social	28.84 ± 9.38	5.11 ± 5.38	*t*_(70)_ = 12.59	<.01
ADOS repetitive behaviors^c^	1.77 ± 1.8	–		
ADOS social	9.11 ± 2.89	–		
ADI‐R repetitive behaviors^d^	5.52 ± 2.74	–		
ADI‐R social	19.73 ± 5.98	–		
ADI‐R communication	9.13 ± 4.42	–		

ADOS, Autism Diagnostic Observation Schedule; ADI‐R, Autistic Diagnostic Instrument‐Revised; SRS, Social Responsiveness Scale.

The ASD group included individuals with Autistic Disorder (*n *= 17), Asperger's Disorder (*n *= 25), and Pervasive Developmental Disorder Not Otherwise Specified (*n *= 8). All ASD and TD participants were required to meet inclusion criteria of Full‐Scale IQ > 70. Means and standard deviations are shown.

a IQ estimate was not available for 3 ASD and 1 TD participants.

b SRS domain scores were available for 41 ASD and 31 TD participants.

c ADOS domain scores were available for 45 ASD participants.

d ADI‐R Parent Interview domain scores were available for 23 ASD participants.

Potential TD participants had no history of neurological disorders, seizure, head trauma with loss of consciousness, mental retardation, pervasive developmental disorders, or any lifetime Axis I psychiatric disorders as assessed using the Structural Clinical Interview for DSM‐IV Axis I Disorder (SCID; First, Spitzer, Gibbon, & Williams, 2002) or Kiddie Schedule for Affective Disorders and Schizophrenia (K‐SADS; Kaufman et al., 1997) by a master's‐level clinician. Additional exclusion criteria for both ASD and TD participants included Full‐Scale IQ < 70 and contraindications to the MRI environment (e.g., ferromagnetic implants or claustrophobia). Intelligence quotient (IQ) for 1 ASD participant was assessed using the Wechsler Intelligence Scale for Children (WISC‐III) (Wechsler, 1991), for 3 ASD participants using the Differential Abilities Scale (DAS) (Elliot, 1990), and for 2 ASD participants using the Wechsler Adult Intelligence Scale (WAIS‐III) (Wechsler, 1997). All other ASD and TD participants were assessed using the Wechsler Abbreviated Scale of Intelligence (WASI) (Wechsler, 1999). The ASD and TD groups were also assessed, using the Social Responsiveness Scale (SRS) (Constantino & Gruber, 2005) as well as other neuropsychological tests that are not part of the current study. Handedness was assessed using the Edinburgh Handedness Inventory (Oldfield, 1971). Socioeconomic status (SES) was assessed using the Hollingshead scale (Hollingshead, 1975).

### 2.2 Main sample

Fifty ASD participants (8–60 years) were matched to 50 TD participants on age, sex, and cognitive ability. Correspondence on these measures was tracked and implemented during recruitment phase of the study. The two groups did not significantly differ in Full‐Scale, Verbal or Performance IQ, handedness, SES, ethnicity (all *p *> .05). Additional participants were scanned but not included in the sample because we could not ascertain their ability to follow instructions on the task (*N *= 4_ASD_; *N *= 3_TD_; <70% accuracy) or due to poor imaging data quality on the basis of visual inspection (*N *= 13_ASD_; *N *= 8_TD_). ASD and TD participants from the initial sample did not differ statistically on age, sex, SES, FSIQ, VIQ, PIQ, or ethnicity. Twenty‐three of 50 ASD participants (and none of the TD participants) were taking psychotropic medication at the time of scan: Stimulants (*n *= 7), Antidepressants (*n *= 25; includes SSRIs and NRIs), Anticonvulsant/Antiepileptic (*n *= 8), Other Antipsychotics (*n *= 6), Benzodiazepine (*n *= 2), Lithium (*n *= 2), MAOI (*n *= 1), and Atypical ADHD medication (*n *= 1) (Note that the total does not equal 23 because some ASD participants took multiple medications).

The proportion of right versus left‐handed (R/L) participants differed between the two groups, with significantly more left‐handed participants in the ASD group (*N *= 50_ASD,_ 26R/24L versus *N *= 50_TD,_ 46R/4L (*X* ^2^ = 19.8413, *p *= 8.414e−06).

All participants had normal or corrected‐to‐normal vision; near‐sighted individuals were fitted with MRI‐compatible corrective lenses. Head cushioning was used to minimize head movement during the scan. The study was approved by the Institutional Review Board at New York State Psychiatric Institute. Prior to consent procedures, the capacity to provide informed consent was determined for all adult ASD participants. Informed consent or assent was obtained from all participants.

### 2.3 Task

Participants performed a spatial stimulus‐response compatibility Simon task (Simon & Rudell, 1967) presented through MRI‐compatible goggles (Resonance Technologies, Inc., Salem, MA, USA) using E‐Prime (Psychology Software Tools, Inc., Sharpsburg, PA, USA). The stimulus consisted of a white arrow presented against a black background subtending 1^o^ visual angle horizontally and vertically. The arrow was presented on the horizontal midline of the goggle display at a constant distance of 4^o^ from the vertical midline. Two feature dimensions of the stimulus varied from trial‐to‐trial: the direction in which the arrow pointed (left or right, i.e., ‘<‘ or ‘>‘) and its location on the screen relative to the vertical midline (either on the left or the right‐hand side). In “congruent” displays the dimensions matched: the arrow pointed to the left and was located on the left side of the screen (or pointed to the right and was located on the right). In “incongruent” displays, the arrow pointed to the left but was presented on the right side of the screen (or it pointed to the right but was presented on the left side of the screen).

On each trial, the arrow was presented for 1300 ms. Participants were instructed to respond as quickly and as accurately as possible to the direction of the arrow while ignoring its spatial location. Participants responded by pressing one button on a response box with their right index finger for the arrow pointing to the “left” or by pressing another button with the right middle finger for arrow pointing to the “right” (if no response was given before time expired (1.3 s), or if response was given within 200 ms of stimulus onset, no response was recorded—the program advanced to the next trial and the trial was coded as “missing”). Left and right responses were equally probable. The stimuli were separated by a jittered inter‐trial interval (ITI) ranging from 4,000 to 8,500 ms (mean* *± SD = 5351.4 ± 842.5 ms), during which time a fixation cross was displayed in the center of the screen. Stimulus presentation and scan acquisition were synchronized to within 20 ms. Stimuli were presented in pseudo‐random order in 3 runs. Each run consisted of 22 congruent and 22 incongruent stimuli. The 50–50 congruency proportion paradigm was selected in order to avoid potential effects of exogenous, bottom up attentional influences associated with rare stimulus onsets. Within each run, each trial (congruent or incongruent) could be preceded with equal probability by either a congruent or an incongruent trial. In total, the experiment consisted of 132 trials (66 incongruent, “I”, and 66 congruent, “C” stimuli). All participants completed a brief training session before initiation of fMRI acquisition to ensure that they understood task rules and could perform the task properly.

### 2.4 Head motion indices

To exclude non‐neural source of variability due to head motion (Friston, Williams, Howard, Frackowiak, & Turner, 1996; Power, Barnes, Snyder, Schlaggar, & Petersen, 2012; Van Dijk, Sabuncu, & Buckner, 2012), we quantified the amount of head motion in the scanner for each participant for each run using two volume‐based summary statistic metrics. In SPM8, realignment of volume images yields a matrix *T*_t_ (Ashburner & Friston, 2004) representing rigid‐body affine transformations (three translational [x, y, z] and three angular rotational parameters [roll, pitch, yaw] of volume images with respect to the reference image [i.e., here, the first volume, the default in SPM8]).

The frame‐wise Displacement (FD_Power_) metric sums differentiated realignment estimates (Power et al., 2012):

(1) $${\text{FD}}_{i}=\left|\Delta d_{ix}\right|+\left|\Delta d_{iy}\right|+\left|\Delta d_{iz}\right|+\left|\Delta \alpha_{i}\right|+\left|\Delta \beta_{i}\right|+\left|\Delta \gamma_{i}\right|,$$

where $\Delta d_{ix}=d_{\left(i-1\right)x}-d_{ix}$ are translational displacements (d) and rotations (α β γ) at frame *i* for the rigid body parameters (Power et al., 2012).

We also calculated Jenkinson's (Jenkinson, Bannister, Brady, & Smith, 2002) root mean squared (RMS) deviation (i.e., error), $d_{\mathrm{RMS}}$,

(2) $$d_{\mathrm{RMS}}=\sqrt{\frac{1}{5}R^{2}Tr\left(M^{T}M\right)+t^{T}t},$$

where $\left(\begin{array}{cc} M & t\\ 0 & 0 \end{array}\right)=T_{j}*A_{j}*T_{0}^{-1}$ is used to compute the 3 × 3 matrix *M* (denoting the center of the image volume) and the 3 × 1 vector *t* (here, *T* is the transformation using the least squares approach in SPM8). Rotational angles for both metrics were projected onto the surface of a sphere of radius 50 mm in order to convert these estimates to arc length in millimeters (Power et al., 2012).

We use mean FD_Power_ values to match cohorts for group‐level neuroimaging inferences; both mean FD_Power_ and relative RMS values per subject are reported in Tables S1 and S2. Across all 100 participants, mean FD values were significantly higher in individuals with ASD relative to TD (Kruskal–Wallis, *p *= .0260).

Finally, we computed parameter estimates of the underlying distribution of head movements, without *a priori* assumptions about the normality of the underlying distribution. Here we simply considered summed (absolute values) differentiated realignment parameters for each frame (“FDi” in mm) following equation* * (1) as a timeseries over the scan and fitted Gamma Probability Distribution (PD) (equation (4) below) for pooled data as well as for each individual participant. The purpose of this additional analysis is twofold: to ensure that individual participants comprising the two neuroimaging cohorts (described below) do not have significant nonlinear signatures in their head movements, as well as to study the relation between movement‐based noise‐to‐signal Fano Factor (FF) and response fluctuation‐based noise‐to‐signal FF in the main sample.

Empirical cumulative distribution functions (eCDFs) of participants’ movement (FDi) data differed significantly (K‐S test, *p *= 1.4075e−100) (Figure S1A). Pooling all 100 participants’ data from the main sample, ASD and TD parameter estimates (PE) differed significantly on noise‐to‐signal levels (i.e., scale (b) or FF) (Figure S1B,C). Examination of individual parameter estimates showed that individuals with ASD had significantly higher noise‐to‐signal ratios relative to TD control participants (ASD median, range: 0.1289 (0.0361–0.5966), TD median, range: 0.1030 (0.0300–0.7933); Kruskal–Wallis, *p *= .0161, using ‘unscrubbed’ data; FF was also significantly worse when using ‘scrubbed’ or motion‐corrected data: Kruskal–Wallis, *p *= .0410). The shape parameter did not significantly differ between ASD and TD participants (Kruskal‐Wallis, *p *> .5).

Neither shape nor scale parameter estimates on ‘scrubbed’ data that entered fMRI/PPI statistical inference (described below) were significant between ASD and TD participants in the ‘genetic’ and ‘cem’ cohorts (‘genetic’ cohort: a (shape) PE: *p *= .4636, b (scale), *p *= .7285; ‘cem’ cohort: a (shape), PE: *p *= .2393, b (scale), *p *= .3369).

### 2.5 Head‐motion matched sub‐sample: Neuroimaging (fMRI and PPI) analyses

We used the following stringent inclusion and exclusion criteria for the sample in the neuroimaging analyses. First, no participant had volume‐to‐volume movement >1 mm or 1 degree. This was checked by plotting and visually inspecting realignment estimates. Second, every participant had FD < 0.2 mm on every run in the experiment (<0.2 mm on average) (Power et al., 2012). Application of the two criteria produced a pool of 83 participants (*N *= 83: *N *= 39_ASD_, *N *= 44_TD_). Third, we used the nonparametric MatchIt program in R (http://gking.harvard.edu/matchit) (Ho, Imai, King, & Stuart, 2007) to form cohorts of participants with and without ASD matched on age, sex, and frame‐wise displacement (FD) so that the difference between groups on FD was <0.004 mm. We opted for this group matching approach because fMRI and connectivity analyses cannot be considered reliable when groups differ by a greater amount (Van Dijk et al., 2012). We tested several available MatchIt algorithms and found that the “coarse exact matching” (cem) algorithm minimized FD to <0.004 mm while also matching on age and sex when forming a cohort that included children, adolescents, and adults (*N *= 20_ASD_, *N *= 20_TD_): age (mea*n *± SD): *N *= 20_ASD_, 22.72 (8.5719) versus *N *= 20_TD_, 23.21 (8.5719) years old (*p *= .2395), sex (M/F): *N *= 20_ASD,_ 19M/1F versus *N *= 20_TD,_ 19M/1F (*X* ^2^ = 0.0, *p *= 1.0), and FD (mea*n *± SD): *N *= 20_ASD_, FD = 0.1223 (0.0368) versus *N *= 20_TD,_ FD = 0.1235 (0.0362) (FD_difference_ = 0.0012 mm; *p *= .9187). Proportion of right versus left‐handed (R/L) participants did not differ between ASD and TD groups (*N *= 20_ASD,_ 16R/4L versus *N *= 20_TD,_ 17R/3L (*X* ^2^ = 0.1732, *p *= .6773).

When forming a children‐only group we found that the “genetic” algorithm served best in minimizing FD below the required amount (*N *= 12_ASD_, *N *= 8_TD_), while matching on age (mean ± SD): *N *= 12_ASD_, 15.74 (3.5804) vs. *N *= 8_TD_, 15.97 (3.6337) years old (*p *= .8769), sex (M/F) *N *= 12_ASD,_ 10M/2F vs. *N *= 8_TD,_ 6M/2F (*X* ^2^ = 0.2083, *p *= .6481), and FD (mean ± SD): *N *= 12_ASD_, FD = 0.1361 (0.0413) versus *N *= 8_TD,_ FD = 0.1369 (0.0358) (FD_difference_ = 0.001; *p *= .9651). Proportion of right versus left‐handed (R/L) participants did not differ between ASD and TD groups (*N *= 12_ASD,_ 10R/2L vs. *N *= 8_TD,_ 6R/2L (*X* ^2^ = 0.2083, *p *= .6481).

No algorithm minimized FD difference sufficiently (i.e., below the 0.004 mm difference threshold) to form an adult group, nor a group of ASD participants “on” or “off” psychotropic medication, while also matching on age and sex. FD values and matched demographic variables for these cohorts are listed in the Supplement.

### 2.6 Analyses: behavioral

#### 2.6.1 Distributional analyses

We empirically estimated parameters of the lognormal distribution from participants’ latencies (reaction time, RT). The lognormal (also Galton; Galton, 1889) distribution describes well the multiplicative nature of natural phenomena (Kello et al., 2010; Limpert, Stahel, & Abbt, 2001) (e.g., perceptual and cognitive functioning represents a product, not addition of, independent, identically distributed random variables) and all four moments are defined for this distribution, although other distributions, such as the Gamma plane (which we use in the trial‐to‐trial fluctuation analyses below), are also suitable.

**Figure 1:**
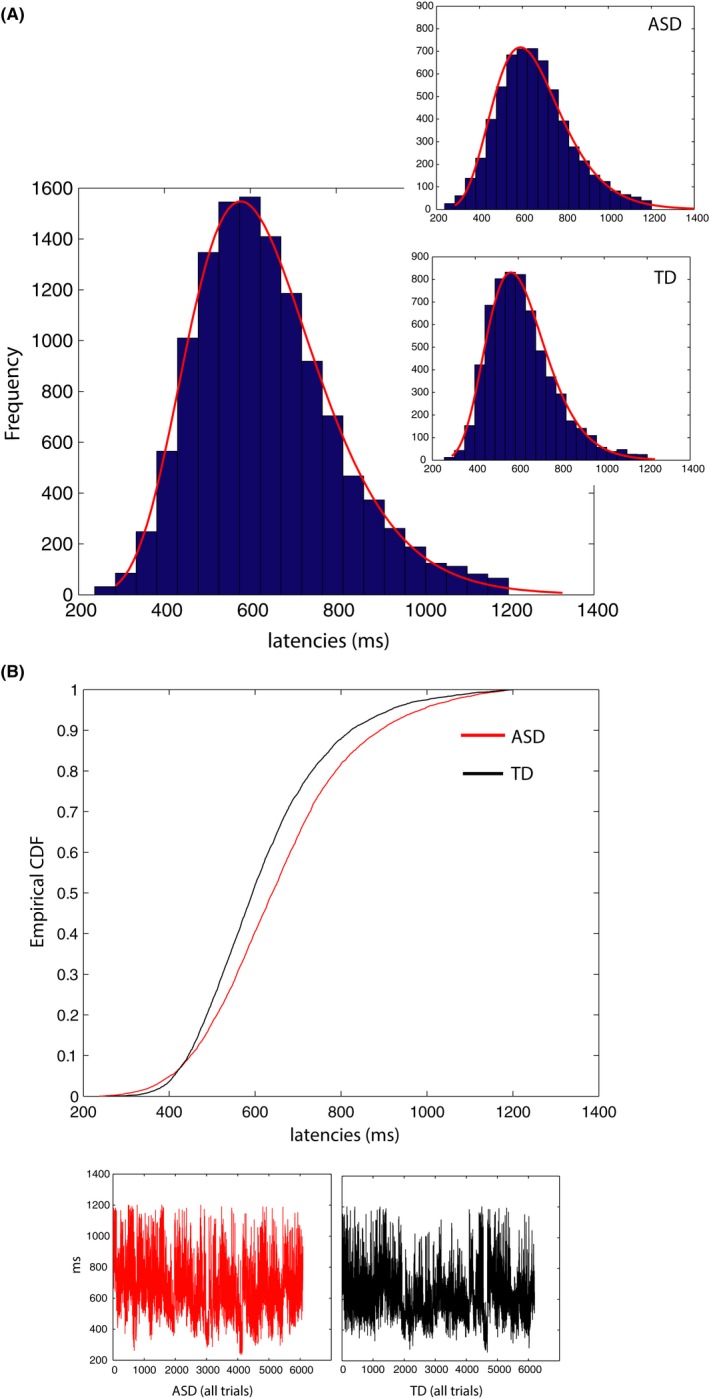
Raw response data shown for all 100 ASD and TD participants. (A). Frequency histograms across participants and all trials, and for 50 ASD and 50 TD participants separately (inset) (Lognormal fits to the data are shown) (B). Empirical Cumulative Distribution Functions (eCDFs) of ASD and TD distributions were significantly different (Kolmogorov–Smirnov test *p *= 1.5760e−42) (Lower panel of b shows raw, unnormalized latencies for all ASD and TD participants)

Note that the assumption of normality usually fails in the case of natural phenomena whose underlying distribution has a heavily skewed tail; this means that parametric tests that assume normality cannot be used with non‐Gaussian data. We used three tests – Kolmogorov–Smirnov, Lilliefors, and Jarque–Bera – to establish that the normality assumption does not hold in our observed data. All three tests (using a total of 12,287 trials; 6,090 for ASD and 6,197 for TD) showed that the latencies are not normally distributed (Kolmogorov–Smirnov, (*p *= .000), Lilliefors (*p *= 1.0000e−03), and Jarque–Bera (*p *= 1.0000e−03) (Figure 1A).

The probability density function (PDF) of the lognormal distribution is defined as follows:

(3) $$y=f\left(x|\mu ,\sigma \right)=\frac{1}{x\sigma \sqrt{2\pi }}e^{\left(-\frac{{\left(\ln\left(x\right)-\mu \right)}^{2}}{2\sigma^{2}}\right)},\;for\;x>0,$$

where μ (mu) and σ (sigma) are the location and scale parameters, which denote the mean (geometric median) and standard deviation of the natural logarithm of *x*, respectively (lognormal distribution is defined for a random variable *x* whose logarithm is normally distributed, $y=\log\left(x\right)$).

We computed the coefficient of variation (a measure of dispersion, $CV=\sqrt{\left(e(\sigma^{2})-1\right)}$), skewness ($\text{skewness}=CV^{3}+3*CV$) and kurtosis ($\text{kurtosis}=CV^{8}+6*CV^{6}+15*CV^{4}+16*CV^{2}$ ) following formulas for lognormal distribution (Strom & Stansbury, 2000). Fano Factor (FF) (Fano, 1947) is a noise‐to‐signal measure, expressed as the ratio of variance over mu: $\text{FF}=\frac{\sigma^{2}}{\mu }$ (FF is large when the variance is large relative to the mean). We estimated parameters after first normalizing latencies (raw RT on each trial) by the maximum latency (*maxRT*) of all trials for each group and condition considered: $\text{Normalized}\;\;\;\text{Latency}=\frac{\text{RT}}{\text{RT}+\text{maxRT}}$. (Note that maxRT was a constant parameter for each participant). All normalized latencies were further normalized by the age of each participant: $\text{Normalized}\;\text{Latency}\;\text{Age}=\frac{\text{Normalized}\;\text{Latency}}{\text{Age}}$. Normalization is necessary because we could not assume that identical raw latencies correspond to similar underlying mental processes in individuals of varied ages across the spectrum of typical and atypical neurodevelopment. Normalization produces a “unit‐less” normalized index between 0 and 1, here denoted as “LA index”, enabling us to consider all responses on a common scale. Higher values on the index denote a shift of mu toward longer latencies (i.e., longer RTs).

We first estimated parameters for all pooled trials for ASD and TD groups. Parameters were then empirically estimated for each of the 100 individual participants for all trials regardless of condition (as well as for both incongruent (interference) and congruent (no interference) conditions). Specifically, in the empirically estimated parameters of lognormal PDF we assessed for the differential effects of task and group, medication intake by ASD participants, and high total ADOS scores (using a median split). Kruskal–Wallis (a nonparametric test of analysis of variance) tested whether individual parameter estimates were different between ASD and TD. We used Statistics Toolbox in MATLAB 8.3 (R2014a) (MathWorks, Natick, MA, USA) for distributional analyses. MATLAB's fitdist estimates the value of sigma as the square root of the unbiased estimate of variance of the log of the data with 95% Confidence Intervals (CIs) for mu and sigma.

Because of the wide age range of participants in our study, we also estimated parameters separately for children and adult participants (< and >21 years old: *N *= 18_ASDChildren_, *N *= 17_TDChildren_ and *N *= 32_ASDAdults_, *N *= 33_TDAdults_). Participants with ASD were median‐split according to the group‐level median (above and below the median score) on the ADOS total score (*N *= 24_ASDHIGHados_, *N *= 21_ASDLOWados_; these equal *N *= 45 with available ADOS scores). (We also performed median split on the ADOS for adult (*N *= 16_ASDadultsHIGHados_, *N *= 17_ASDadultsLOWados_) and children (*N *= 6_ASDchildrensHIGHados_, *N *= 6_ASDchildrenLOWados_) participants. We further investigated potential differences between individuals with ASD who were currently “on” or “off” psychotropic medication (*N *= 23_ASDMed_, *N *= 27_ASDNoMed_) relative to TD controls. (We also performed separate analyses for children and adults on medication (children: *N *= 8_ASDChildMed_, *N *= 10_ASDChildNoMed_) and adults (*N *= 15_ASDAdultMed_, *N *= 17_ASDAdultNoMed_) relative to TD children (*N *= 17) and TD adult (*N *= 33) participants.

With regard to medication analyses, we further considered the role of different medication classes on parameter estimates. Children and adults with ASD were prescribed different medications, and different combinations of medications (note that these analyses were possible only for a subset of participants who could be grouped under similar medication classes; due to the small N for each subgroup we do not examine noise‐to‐signal ratio for these subgroups). Out of 8 ASD children on medication, *N *= 3 children were on antidepressants in combination with a stimulant, and *N *= 2 were on medication other than antidepressant or stimulant (anticonvulsant or antipsychotic). Out of 15 adults with ASD on medication, *N *= 4 were on antidepressants and another medication (not a stimulant), *N *= 3 were on antidepressants alone (no other medication), and *N *= 4 were on medications other than antidepressants or stimulants (anticonvulsants, atypical ADHD medication, or MAOI). The purpose of this latter grouping for adults was to form a “medication” comparison group against all other groups, which were confined to antidepressants, or antidepressant in combination with another medication class.

##### 2.6.1.1 Normalized peaks analyses

The conventional latencies analyses (above) allow study of how ASD individuals differ from TD individuals, not only on the parameters of mu and sigma (mean and standard deviation), but also, for example, in the levels of noise‐to‐signal ratio (FF). Here we probe further whether subtle differences in trial‐to‐trial fluctuation of peak latencies over the entire experiment are detectable using the Gamma parameter plane. Gamma is a family of distributions, ranging from Gaussian (normally distributed, symmetrical) to Exponential (more random and noisy, heavy‐tailed).

Here our goal was to investigate the character of subtle fluctuations (quantitative character of peaks and troughs) in the two groups. Gamma PDF is especially suitable for this type of analysis because prior work shows that parameter estimates of individuals with neurodevelopmental (as well as neurodegenerative) disorders fall closer to the exponential range of the Gamma PDF, whereas TD individuals’ estimates are closer to Gaussian ranges (Torres et al., 2016). Hence, the Gamma parameter plane allows study of phenomena without assuming a specific distribution (Torres, 2011, 2013).

The probability density function (PDF) of the Gamma distribution is defined as:

(4) $$y=f\left(x|a,b\right)=\frac{1}{b^{a}\Gamma \left(\cdot \right)}x^{a-1}e^{\frac{-x}{b}},\;for\;x>0$$

where Γ(·) is the Gamma function (modeling sums of exponentially distributed random variables), and *a* and *b* are its shape and scale parameters. Large *a* values mean that the distribution is closer to the normal (Gaussian) distribution, whereas smaller values indicate a shift toward a more Exponential distribution. The scale, *b*, parameter of the Gamma distribution is equal to the Fano Factor (FF). MATLAB estimates 95% CIs for Gamma fits using maximum likelihood estimation procedure.

We estimated Gamma parameters after first extracting peaks (maxima) from the entire timeseries of all latencies over all runs (raw peaks for all participants per group are shown in the right panel of Figure 5A). We then normalized the waveform by dividing each peak by the mean of the surrounding minima, for each individual (or subgroup) considered: $\text{Normalized}\;\text{Peaks}=\frac{\text{RT(peak)}}{\text{RT(peak)}+\text{RT}\left(\text{average}\left(\text{surrounding}\;\;\text{minima}\right)\right)}$ .

(For additional methodological details on “micro‐movements”, please see (Torres et al., 2016)). Peaks were separated as “above” or “below” the median. Results from both sets are reported; we focus on “above” the median peaks set in this work (the “below” median set would represent small movements or “tremors” requiring higher sampling resolution to study accurately). Similar to the lognormal analyses above, all normalized peaks were further normalized by the age of each participant: $\text{Normalized Peaks Age}=\frac{\text{Normalized Peaks}}{\text{Age}}$ . This normalization produces a “unit‐less” normalized index between 0 and 1, here denoted as “NormalizedPeaksAge index” (NPA). We first fitted Gamma parameters over the entire group (50 ASD and 50 TD participants), and then for each individual participant. We used polyfit to examine the relation between individual estimates of shape and scale, plotting residuals to help visualize deviation from linearity for ASD and TD (a single equation fit data of all 100 participants). In order to increase power for this analysis when fitting subgroup data, we combined normalized peaks as a vector.

### 2.7 Analyses: Imaging

#### 2.7.1 Image acquisition parameters

Functional scans were acquired on a 3T GE Signa EXCITE scanner (GE Medical Systems, Milwaukee, WI) with a standard quadrature GE head coil. A T1‐weighted sagittal localizing scan was performed to position the subsequent axial‐oblique fMRI volumes parallel to the anterior commissure‐posterior commissure (AC‐PC) plane. Using a T2*‐weighted single‐shot gradient echo planar imaging (EPI) pulse sequence [2,200 ms repetition time (TR), 30 ms echo time (TE), 90 degree flip angle, 240 mm field of view (FOV), 64 × 64 matrix size, and 62.5 kHz receiver bandwidth] we acquired 34 contiguous interleaved whole‐brain axial‐oblique slices with 3.5 mm thickness and 3.75 × 3.75 mm in‐plane resolution. The first 6 volumes of each run were “dummy volumes” and were discarded to allow the scanner to stabilize. Each experimental session consisted of 3 functional runs, for a total of 140 volumes per run. Each run lasted 5 min, 21 s, for a total EPI scan time of 16 min, 3 s.

#### 2.7.2 fMRI image preprocessing

The preprocessing of images was performed using SPM8 (http://www.fil.ion.ucl.ac.uk/spm) and in‐house custom code running MATLAB 8.3 (R2014a) (Mathworks, Natick, MA). Functional images were slice‐time corrected and realigned to the first image using least squares rigid transformation. Head motion‐induced spikes in the time series were excised (“censored”), using SPM8's ArtRepair function which then interpolated over censored data using adjacent volumes (i.e., “scrubbing”; Power et al., 2012). We used 119 volumes per run (85%) as a minimum requirement for the uncensored number of volumes remaining after scrubbing. The images were then normalized to the standard EPI T2* MNI template with a resampled voxel size of 3 × 3 × 3 mm^3^. The data were spatially smoothed by convolving the images with an 8 mm full‐width at half maximum (FWHM) Gaussian kernel. High‐pass filtering was applied to the time series to remove low‐frequency drift (cutoff frequency of 1/128 Hz via discrete cosine transform). Serial correlations in the fMRI time series and in the regressors of the design matrix were removed using the first‐order autoregressive model AR (1) and the restricted maximum likelihood algorithm.

#### 2.7.3 fMRI time series modeling

We modeled fMRI timeseries for each participant (“first‐level”) using several general linear models (GLM) in SPM8 (http://www.fil.ion.ucl.ac.uk/spm) running MATLAB 8.3 (R2014a) (Mathworks, Natick, MA). In GLM1, event‐related variable‐epoch regressors (box‐car functions convolved with a canonical hemodynamic response function (HRF) and its first temporal derivative) corresponded to the stimulus onsets with duration equal to the reaction time on that trial (Grinband, Wager, Lindquist, Ferrera, & Hirsch, 2008). Neural activity scales with reaction times (RTs); by modeling RTs as an index of neural processing on each trial for each participant, we minimized the potential effects of effort or sustained attention on hemodynamic activity across trials (Boynton, Engel, & Heeger, 2012). For GLM1, we constructed single‐subject contrasts to isolate brain activity as a function of the presence or absence of cognitive interference, creating 2 task‐related regressors: congruent (C) and incongruent (I) trials.

GLM2 and GLM3 were conducted post hoc. In GLM2, we searched for differences across trials (regardless of condition) between the ASD and TD groups. One variable‐epoch regressor corresponded to stimulus onsets equal to the RT on that trial, as for GLM1, with the exception that GLM2 did not take into account the interference level on each trial. In GLM3, we assessed whether BOLD differs in ASD either as a function of the decision to respond to a stimulus, or as a function of the ‘refractory’ phase immediately after the response, during which time the stimulus was still visible on the screen (i.e., up to 1300 ms). We constructed GLM3 with two regressors that indexed these two phases: the 1st part of the trial was identical to the main GLM1 model (time‐on‐task), and the 2nd part of the trial following the decision to respond with the button‐press. In preliminary pilot testing, we ran 2 versions of all models: one using trials with correct‐only behavioral responses, and another using all trials (correct and incorrect). No differences were revealed between these two versions, and therefore, we report results from the models that consider all trials.

For all models, nuisance regressors included missing responses and six rigid‐body realignment parameters (three translational coordinates and three rotational angles). Group‐level (“second‐level”) analyses detected task‐related activity within and between diagnostic groups. Mean‐centered age and sex were entered as covariates of noninterest in all group‐level analyses.

#### 2.7.4 Functional connectivity: psychophysiological interaction

We assessed the psychophysiological interaction (PPI) between the brain region of interest and its time course (physiological component) depending on the particular experimental condition (psychological component; Friston et al., 1997). This analysis reveals whether other brain regions follow a time course similar to that of the reference region, a measure of functional connectivity; we opted for the seed‐to‐whole‐brain approach. For each seed separately, we extracted the activation time course (first eigenvariate), deconvolved it to estimate the neural time course, multiplied by the vector of the contrast (trials with high‐interference were set to 1 and low‐interference trials were set to −1) and lastly convolved with the HRF to produce the PPI time course (The psychological component—the vector of the experimental contrast—was also convolved with the HRF). This resulted in new regressors: the PPI term, the physiological variable, and the psychological variable. We conducted the first‐level analyses, using PPI as the main regressor and psychophysical and physiological terms as regressors of noninterest (this ensures orthogonality of the PPI output; O'Reilly, Woolrich, Behrens, Smith, & Johansen‐Berg, 2012). A high‐pass filter (1/128 Hz) removed low‐frequency drifts in the data. In addition, we included six motion parameters as nuisance regressors in the PPI model.

We used a 6 mm radius sphere centered on the peak of each of the nine PPI seed coordinates derived independently of the fMRI task, using a combination of literature‐review based and anatomical criteria, as follows. Seven regions of interest (ROI) coordinates belonged to regions that were previously established to participate in diverse cognitive demands, with MNI centers of mass (peaks) following (Woolgar, Hampshire, Thompson, & Duncan, 2011): a unified Anterior Cingulate/pre‐supplementary motor area, ACC/pre‐SMA (0, 23, 39), left and right inferior frontal sulcus, IFS (± 38, 26, 24), left and right anterior insula/frontal operculum, AI/FO (± 35, 19, 3), and left and right intraparietal sulcus, IPS (± 35, −58, 41). Left and right cerebellar seeds were also chosen, focused on peak coordinates of lobule VII (Crus I) (left: −37, 73, −28, right: 37, −72, −30) using the Anatomy Toolbox Atlas (Eickhoff et al., 2005, 2007) (we focus on this lobule because it contains nearly half of the estimated gray matter by volume in the cerebellum (Diedrichsen, Balsters, Flavell, Cussans, & Ramnani, 2009)).

In summary, we tested for a psychophysiological interaction to assess in each participant the interference‐sensitive coupling between a priori regions of interest and the rest of the brain. The psychological factor was the interference effect as encoded by our stimulus types; the physiological variable was the time course at each ROI seed. In essence, this analysis models changes in (linear) coupling—between the seed regions and all other brain regions—with changes in cognitive interference. This interference‐specific coupling effect was estimated for each subject, thus enabling us to compare the coupling change in the two groups at the between‐subject level, using the summary statistic approach.

#### 2.7.5 Statistical inference: fMRI and PPI

Nonparametric permutation tests were performed for all group‐level (within‐ and between‐group) inferences using individual participants’ (first‐level) maps with 5000 permutations (except as noted below for within‐group inferences) using ‘randomise’ (Winkler, Ridgway, Webster, Smith, & Nichols, 2014) command in FSL (FMRIB Software Library v2.9) for each inference. The randomization process empirically generates the sampling distribution under the null hypothesis that is used for significance testing. Within‐group analysis involves random flipping of the sign of each participant's data (for a one‐sample t‐test, strictly speaking, data are not subjected to a permutation procedure but to a “sign flip” (Winkler et al., 2014). The maximum number of combinations in the within‐group inference is denoted as 2^N^ (N is the sample size) (for our main analyses, these are 2^20^ = 1,048,576 (20 ASD or 20 TD) for CEM cohort and 2^12^ = 4,096 (12 ASD) and 2^8^ = 256 (8 TD) for the ‘genetic’ cohort). Between‐group analysis (two‐sample t‐test) involves both random permutation (re‐assignment of group labels to participants; here, ASD and TD) and sign flipping, drawing a random sample 5000 times from a maximum of possible combinations, as follows. The maximum number of possible permutations ([n1 + n1]!/[n1!*n1!], with n1 and n2 designating our two diagnostic groups) was 125,970 for the children's ‘genetic’ cohort (20!/[12!*8!]), and 1.3785e+11 (40!/[20!*20!]) for the ‘cem’ cohort. Brain‐behavior inferences were conducted as a between‐group analysis. In particular, for the two groups of ASD participants split according to the “high” versus “low” behavioral uncertainty during the scan using median score, 184,756 was the maximum number of possible permutations (20!/[10!*10!]).

Group‐level inferences were corrected for multiple comparisons using Family‐Wise Error (FWE)‐corrected probability P value of 0.05 with a Threshold‐Free Cluster Extent (TFCE). FSL's ‘randomise’ produces output as “1‐P”, meaning that “1” is the highest value possible. We used SPM‐based xjView to present BOLD activation and PPI maps against the MNI152 template (http://www.alivelearn.net/xjview). Brain region definitions at MNI‐based coordinates were based on the probabilistic atlas in SPM8's Anatomy Toolbox (Eickhoff et al., 2005, 2007).

In summary, we used multiple analytic techniques to investigate the nature of neural processing during an interference task in individuals with ASD relative to TD controls. We used a factorial design with one within‐subject factor (presence or absence of interference) and one between‐subject factor (autism vs. typically developing participants). First, we tested for differences in empirically estimated parameters of latencies and their fluctuations, testing specifically for group differences in noise‐to‐signal measures over the task. We then applied GLM analyses to brain activation in a subset of participants from the total sample, again testing for main effects of task (and decision stage) and group differences. Third, we tested specific hypotheses about dysfunctional connectivity in autism using a psychophysiological interaction analysis. In brief, this analysis looked for interference‐specific changes in coupling with key seed regions—and differences in these connectivity effects between groups (Note that here we also defined groups in a data‐driven manner using empirically estimated parameters (i.e., by separating individuals into ‘high’ versus ‘low’ subgroups using the median score on shape and scale) on task during the scan). Finally, at the between‐subject level, we assessed for differences in subject‐specific variables such as medication intake, noise‐to‐signal levels in spontaneous movements during the scan, and clinical measures.

## 3 Results

**Figure 2:**
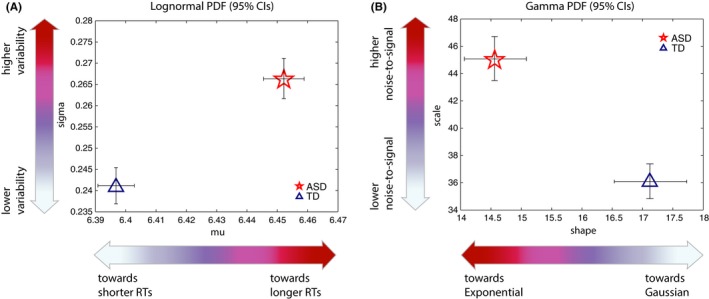
Parameter estimates for latencies for ASD and TD participants, for Lognormal (A) and Gamma (B) distributions. (A) Empirically estimated parameter estimates on the Lognormal parameter plane show significant separation between ASD and TD on both mu and sigma parameters. As a group, ASD had longer latencies (larger mu; shift to the right on the x‐axis) and higher standard deviation (larger sigma; shift upwards on the y‐axis). (B) Empirically estimated parameter estimates on the Gamma parameter plane show significant separation between ASD and TD on both shape and scale parameters. As a group, ASD distribution had a more random shape parameter (lower shape value; shift to the left on the x‐axis toward more Exponential ranges) and higher scale (higher or ‘worse’ noise‐to‐signal levels; shift upwards on the y‐axis). 95% Confidence Intervals are shown

We first considered frequency histograms across all trials for ASD and TD participants (Figure 1A, insets). A two‐sample Kolmogorov–Smirnov test of empirical cumulative distribution functions (eCDFs) showed that ASD and TD participants’ data come from different distributions (K‐S test, *p *= 1.5760e−42) (Figure 1B). Parameter estimates of mu and sigma on the Lognormal parameter plane revealed nonoverlapping 95% CIs for ASD and TD when considering all raw latencies regardless of condition (Figure 2A) (Note that Figure 2B for shape and scale on the Gamma plane; non‐overlapping 95% CIs for ASD and TD). ASD distribution was shifted toward longer latencies, to the right on the x‐axis in Figure 2A (μ_ASD_ = 6.45214 [6.44544, 6.45883 95% CIs], μ_TD_ =  6.39682 [6.39082, 6.40283 95% CIs]) and was characterized by a higher standard deviation (an upwards shift on the y‐axis) (σ_ASD_ = 0.266328 [0.261681, 0.271144 95% CIs], σ_TD_ =  0.241117 [0.236946, 0.245439 95% CIs]). In addition, the ASD distribution was characterized by significantly higher coefficient of variation, CV (CV_ASD_ = 0.2711 [0.2662, 0.271144 95% CIs] versus CV_TD_ =  0.2447 [0.2403, 0.2492 95% CIs]). Noise‐to‐signal ratio (Fano factor, FF) was significantly worse for ASD: 0.011 [0.0106, 0.0114 95% CIs] compared to TD: 0.0091 [0.0088, 0.0094 95% CIs] (higher values of FF denote worse noise‐to‐signal ratio). Skewness and kurtosis also differed significantly in the ASD distribution (Skewness_ASD_ = 0.8333 [0.8175, 0.8497 95% CIs] vs. Skewness_TD_ =  0.7486 [0.7348, 0.763 95% CIs], Kurtosis_ASD_ = 1.2596 [1.2115, 1.3106 95% CIs] vs. Kurtosis_TD_ =  1.0128 [0.9752, 1.0527 95% CIs]).

**Figure 3:**
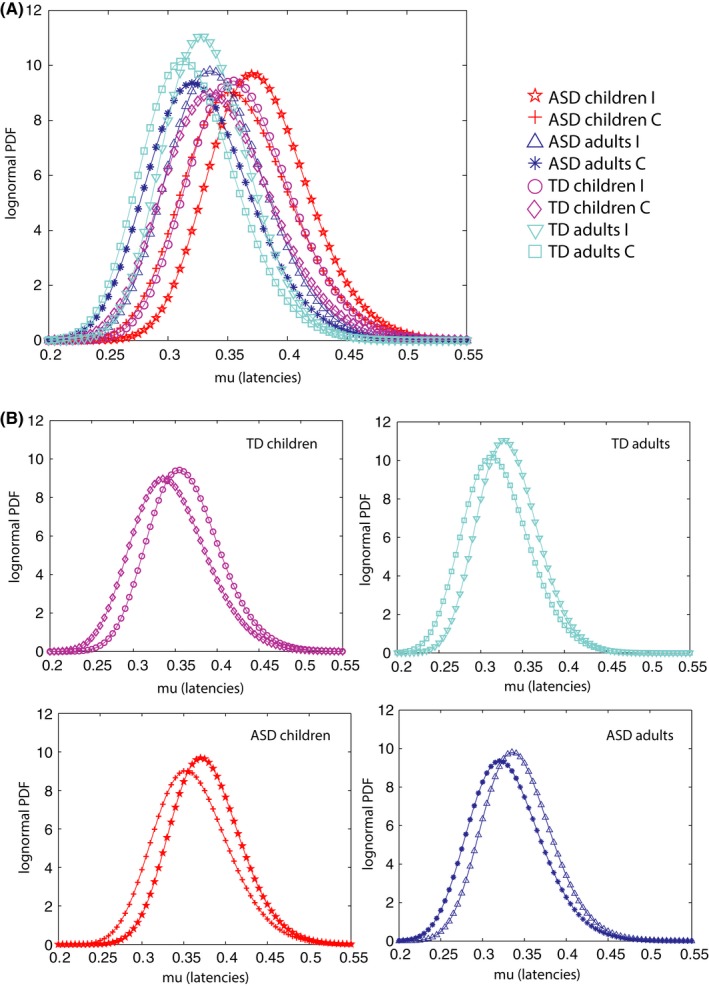
Lognormal Probability Density Functions (PDFs) for each level of interference on the task. (A) Data shown for ASD and TD children and adults, for Incongruent (I) and Congruent (C) trials. (B) Lower panels show data in A separated by age. For all subgroups, higher interference trials produced PDFs that were shifted toward the right on the x‐axis (toward longer mu)

When examining individual parameter estimates (on the Lognormal distribution parameter plane) we found significant differences in mu in individuals with ASD relative to TD participants (Kruskal–Wallis, *p *= .044). Figure 3A shows parameter estimates for I and C trials, for children and adults with and without ASD (Figure 3B presents results separately for each subgroup). Across all groups considered, we found that individual participants had a longer mu on incongruent (I) trials relative to congruent (C) trials, but differences were marginally not significant between diagnostic groups; therefore we focus on characterizing between‐group differences on all trials regardless of interference.

**Figure 4:**
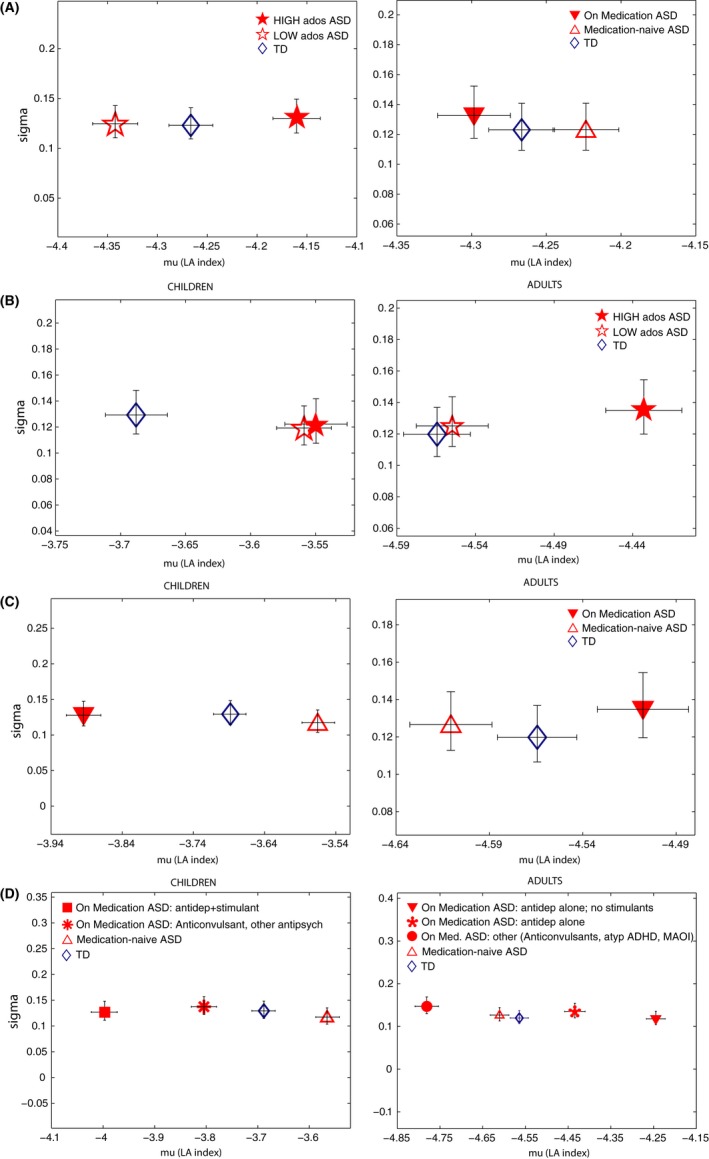
Parameter estimates on the Lognormal plane for ASD and TD subgroups (normalized latencies analysis). (A) Data shown for all participants regardless of age. Left panel shows individuals with ASD with High versus Low scores on ADOS total relative to TD participants, and right panel shows individuals with ASD who were “on” or “off” (medication‐naïve) psychotropic medication at the time of the scan, relative to TD participants. (B–D) show data separately for children (left panels) and adults (right panels) with ASD relative to TD children and adults in the sample. (B). Both children and adults in the high ADOS subgroups had significantly higher mu (shift toward the right on the x‐axis) on the normalized latencies index relative to TD controls. Note that in the case of children with ASD, worse parameters were found regardless of ADOS severity (nonoverlapping 95% CIs relative to TD children). (C). Parameter estimates for ASD individuals currently “on” and “off” psychotropic medication. Parameter estimates (mu) for ASD children on medication were significantly lower relative to both ASD children off medication and TD children. In the right panel, a reverse situation is observed for adults: mu parameter estimates are highest for ASD adults “on” medication. (D). Here we consider medication status information in (C) but now separated by different medication classes. 95% Confidence Intervals are shown

Examination of individual estimates on the Lognormal distribution parameter plane using the normalized LA index across all trials considered (note that higher or “worse” parameter estimates correspond to less negative values, toward the right of the x‐axis; ordering relative to TD group is identical as in Figure 2A) by subgroup revealed that individuals with ASD with the highest deficits on ADOS scores (in the “high” median‐split group) had significantly longer mu, with nonoverlapping 95% CIs relative to TD individuals (Figure 4A, left panel). This relation held when we separately examined children and adults with ASD and matched TD controls (Figure 4B). However, age‐related differences emerged when considering also ASD participants in the low median‐split group.

We found that ASD adults in the high ADOS subgroup had a significantly worse mu parameter, with nonoverlapping 95% CIs relative to both adult TD controls and adults with ASD in the lower median‐split group (Figure 4B, right panel). ASD adults in the high median‐split ADOS subgroup also had a significantly worse noise‐to‐signal ratio, FF, relative to TD adults (Kruskal–Wallis, *p *= .0112). No difference in FF was detected between low ADOS ASD adults versus TD adults (Kruskal–Wallis, *p *= .2823).

Children with ASD, on the other hand, had a significantly worse mu regardless of the severity of their ADOS scores (nonoverlapping 95% CIs for both “low” and “high” ADOS subgroups relative to TD controls, Figure 4B, left panel). No significant differences in FF were detected between children grouped by their ADOS scores relative to TD children. In particular, ASD children in the high ADOS subgroup did not differ on FF relative to TD children (Kruskal–Wallis, *p *= .3627); although ASD children in the low ADOS subgroup had a lower FF relative to TD children, this difference was marginally not significant (Kruskal–Wallis, *p *= .0587).

### 3.1 Medication status

When considering the role of medication status on parameter estimates in all participants regardless of age, medication‐naïve ASD individuals showed the worst mu, worse than TD controls and ASD participants who were currently taking medication (nonoverlapping 95% CIs: Figure 4A, right panel). Examination of parameter estimates for children and adults separately revealed that adults with ASD who were taking medication had the worst mu relative to both TD controls and adults with ASD who were not on medication (nonoverlapping 95% CIs: Figure 4C, right panel). ASD adults taking medication had significantly higher FF relative to TD adult controls (Kruskal–Wallis, *p *= .0140) (no difference in FF was detected between ASD adults “off” medication and TD adults: Kruskal–Wallis, *p *= .4185).

An opposite pattern was found in children with ASD: those who were medication‐naïve showed the worst (longest) mu relative to TD children, as well as relative to ASD children who were taking medication and who had shortest mu (nonoverlapping 95% CIs: Figure 4C, left panel). Although no difference in FF was detected between ASD children taking medication versus TD children (Kruskal–Wallis, *p *= .3513), FF was significantly lower in ASD children off medication versus TD children (Kruskal–Wallis, *p *= .0272).

We next examined estimates for ASD children (N_ASDChild_ = 8) and adults (N_ASDAdults_ = 15) separately as a function of specific medication class (or combination of medication classes). ASD Adults on antidepressants and another drug class (but no stimulants) revealed the worst mu, followed by antidepressants alone (no other drug) (nonoverlapping 95% CIs: Figure 4D, right panel). In contrast, ASD adults who were on medication other than antidepressants (e.g., anticonvulsants) had shorter mu relative to TD (nonoverlapping 95% CIs: Figure 4D, right panel). For children with ASD, we found that the pattern in Figure 4C (left panel, overall longer mu for medication‐naïve ASD children) was consistent no matter how we grouped medication classes (Figure 4D, left panel): children on a combination of antidepressants and stimulants, as well as children separately on an anticonvulsant and other antipsychotic showed shorter mu parameter estimates relative to medication‐naïve ASD children (nonoverlapping 95% CIs).

### 3.2 Stochastic results

In the empirical estimation of parameters of RT (latencies) above, we showed that ASD individuals differed significantly from TD individuals on the parameters of mu and sigma (mean and standard deviation), but also importantly for some subgroups, on the noise‐to‐signal ratio (FF). These findings warranted a closer look at parameter estimates of individuals comprising the ASD group (relative to TD group) on the Gamma plane, whose family of distributions ranges from nearly Gaussian to more random and noisy Exponential distribution (but without requiring an *a priori* assumption of any particular distribution). Here we report subtle, significant differences in the *fluctuation* of trial‐to‐trial variability of responses using the Gamma parameter plane.

**Figure 5:**
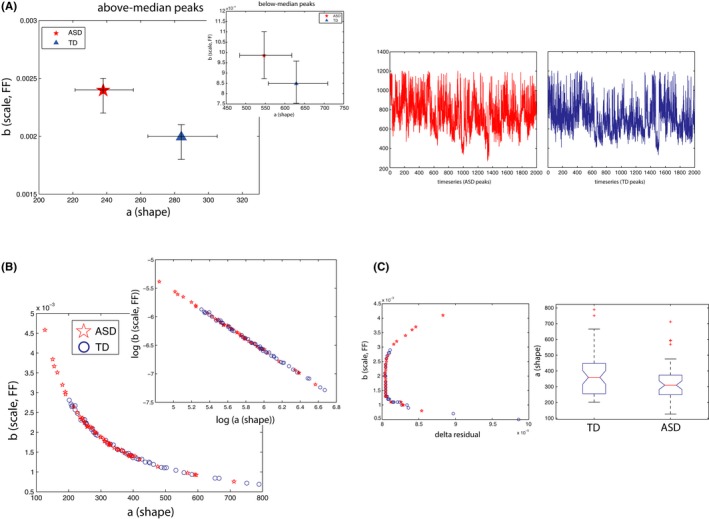
Parameter estimates for trial‐to‐trial fluctuations on the Gamma parameter plane for ASD and TD (normalized peaks analysis). (A) Significant separation between ASD and TD on both shape and scale parameters is shown for the above‐median peaks, with ASD distribution tending toward more random, Exponential ranges on the Gamma plane (to the left on the x‐axis; shape parameter), and toward higher noise‐to‐signal ranges (upwards on the y‐axis; scale parameter or fano factor, FF) (Inset: “below‐median” peaks set shows a similar pattern) (Right panel shows raw peaks for ASD and TD participants). 95 % Confidence Intervals are shown. (B) Gamma fits to individual participants’ data (50 ASD and 50 TD). Here we show a power‐law relation for all participants between shape and scale parameters. Lower values on the shape parameter are located higher on the noise‐to‐signal (scale) parameter (inset: same data shown on double logarithmic axes). (C) The left panel shows a plot of the delta residuals from a polyfit in (b); although deviation from fits was not significantly different for ASD versus TD, we can observe extreme values for ASD participants at the highest FF value range (red stars). The right panel shows that individual fits of the shape parameter were significantly lower for ASD relative to TD participants (Kruskal‐Wallis, *p *= .04)

**Figure 6:**
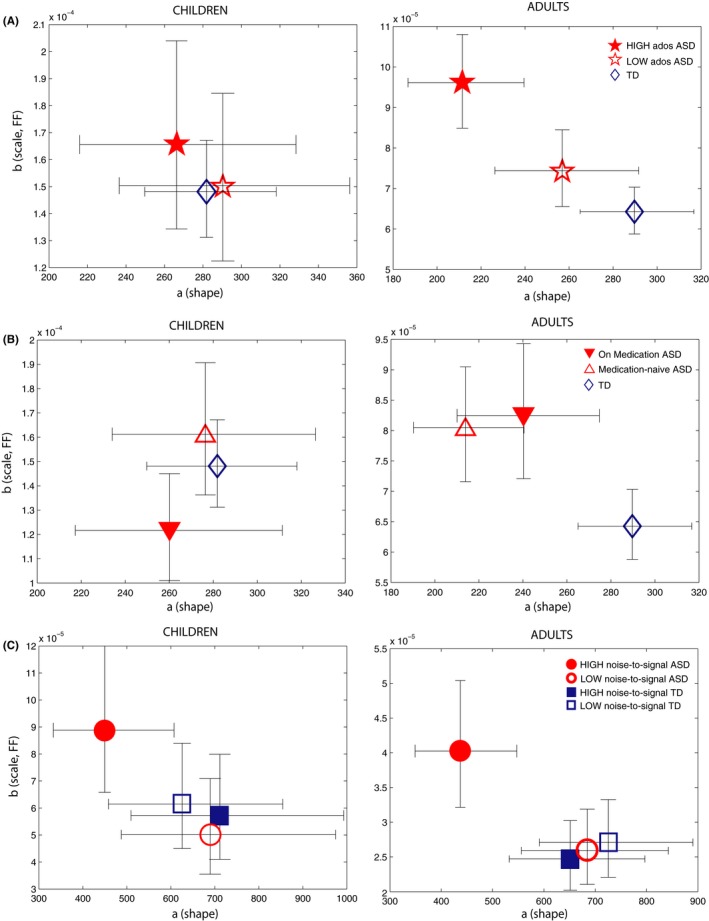
Parameter estimates for fluctuations on the Gamma parameter plane for ASD and TD subgroups. (A, B, and C) consider Gamma parameters of children (left panels) and adults (right panels) separately as a function of ADOS deficits, medication intake, and levels of noise‐to‐signal in involuntary movements during the scan. (A) Adults with ASD with highest ADOS deficits present the most random and noisy parameter estimates relative to TD controls (no difference was found in children's group; left panel). (B) Adults with ASD, whether “on” or “off” medication, present the most random and noisy Gamma estimates (no difference was found in children's group; left panel). (C) Adults with ASD with the “worst” levels of noise‐to‐signal (“HIGH noise‐to‐signal”) in their head movements during the scan present the worst Gamma estimates in their response fluctuations during the scan (no difference was found in children's group; left panel). 95% Confidence Intervals are shown

Figure 5A shows parameter estimates on the Gamma plane for ASD and TD participants. We focus on the “above” median peaks in this work (“below” peaks, inset, would likely represent fluctuations on the order below sensitivity of our sampling resolution, and hence we do not focus on these here. One exception was when we linked FF from head movements during the scan to stochastic signatures of response fluctuations during the same time; there only “below” peaks show significant differences between groups (Figure 6C) which we report).

As expected, parameter estimates in the ASD group were shifted to the left on the x‐axis (shape parameter) and upwards on the y‐axis (scale or noise‐to‐signal parameter), while TD controls were to the right and downwards (Figure 5A). A leftward shift of the ASD distribution, toward Exponential range on the Gamma, indicates a more random structure of the underlying distribution. In contrast, TD distribution reveals a more “normal” or Gaussian structure (nonoverlapping 95% CIs). Furthermore, the ASD distribution is significantly shifted upwards, revealing higher Noise‐to‐signal on the b (scale) parameter, which is also FF, relative to the TD distribution (nonoverlapping 95% CIs) (right panel in Figure 5A shows raw peaks as a timeseries for ASD and TD).

Before doing subgroup analyses, we first fitted Gamma PD to empirically estimate shape and scale parameters individually for each of the 100 participants (Figure 5B, inset shows plot on the double logarithmic axes for all individual fits). We find a general linear (power‐law) relation between the shape and scale parameters, with the lower values on shape (leftward, toward more Exponential distributions) having high values on the scale parameter (upwards, toward “worse” noise‐to‐signal ratio, Fano Factor). The power fit is given by $f\left(x\right)=0.7121*x^{-1.043}$ (Goodness‐of‐fit: SSE: 6.247e−08, DFE = 98, R‐square: 0.9987, Adjusted R‐square = 0.9987, RMSE: 2.525e−05). Visual inspection reveals that some individuals with ASD are especially high on the FF parameter (y‐axis). Although a polyfit did not reveal between‐group differences in residual errors in FF to the fit (Figure 5C), a significant difference was found between individual estimates of the shape parameter (i.e., the left‐ vs. right‐shift between ASD and TD distributions seen in Figure 1A) (Figure 5C, right panel; Kruskal–Wallis, *p *= .04). To emphasize, this result indicates that not only the ASD distribution overall is more “random”, further away from the more normative Gaussian distribution (Figure 5A), but moreover, this difference between ASD and TD participants is detectable when fitting data from each individual participant (Figure 5C).

We then explored differences in these parameters for ASD individuals subgrouped by higher (vs. lower) ADOS TOTAL scores, as well as for individuals “on” or “off” medication relative to TD controls, separately for children and adults (fits based on NPA index). We found that both shape and scale (FF) parameters characterizing fluctuation patterns systematically worsened with the degree of ADOS deficits in adults with ASD (Figure 6A, right panel), with significantly worse stochastic signatures in adults with the highest ADOS deficits (nonoverlapping 95% CIs) relative to TD controls.

With regard to medication subgroup analyses, however, although ASD adults taking medication at the time of the scan showed significantly higher FF in their fluctuations relative to adult TD controls (nonoverlapping 95% CIs, Figure 6B, right panel) these results did not differ relative to medication‐naïve ASD adults. Thus, we found significantly worse stochastic signatures in adults with ASD regardless of medication status, relative to TD adults.

Finally, we explored the relation between noise‐to‐signal (FF) levels obtained from spontaneous head movements during the scan with stochastic signatures of response fluctuations (Figure 6C, right panel). Unlike previous analyses above, we found significant between‐group differences when examining “below” (rather than “above”) median peaks. Here, we found that ASD adults who had the worst (“high”) head movement noise‐to‐signal ratios (FF) also had significantly worse noise‐to‐signal FF in their “below‐peaks” fluctuations relative to low noise‐to‐signal ASD as well as high noise‐to‐signal TD subgroups (nonoverlapping 95% CIs) (in addition, high noise‐to‐signal ASD subgroup was also significantly different relative to the low noise‐to‐signal TD group on shape parameter [nonoverlapping 95% CIs]).

We did not detect a difference in empirically estimated distributional parameters of fluctuations in children subgroups with and without ASD relative to TD children, either when groups were examined by medication status or degree of clinical deficits (overlapping 95% CIs in Figure 6A and B, left panels). Notice that although children with ASD showed a systematic shift whereby increased FF on movement revealed the worst shape and scale (FF) parameter estimates on response fluctuation analysis (“below‐peaks” fluctuation), this trend was not significant (overlapping 95% CIs; Figure 6C, left panel). We did not observe a significant separation between children subgroups in part because fewer trials were available for these analyses.

The findings from the stochastic analyses examining subtle fluctuations in behavior during the scan are overall consistent with our earlier analyses that considered all latencies as a timeseries (Figure 2), as well as were able to reveal new information. First we found that the ASD distribution characterizing fluctuation of response patterns was significantly “worse” both on the shape as well as scale, the noise‐to‐signal (FF) parameter, indicating both higher noise‐to‐signal levels in ASD as well as a less Gaussian distribution (i.e., closer to a more random, Exponential distribution) overall (Figure 5A). Importantly, individual parameter estimates of fluctuation patterns were significantly more random and noisy (i.e., toward Exponential distribution) for ASD individuals (Figure 5C). When considering subgroup analyses, adults with ASD with higher clinical deficits showed the worst noise‐to‐signal ratio (FF) relative to TD controls. Finally, we linked FF from spontaneous head movements to FF in response fluctuations: adults with ASD with the worst noise‐to‐signal levels on movement also had the worst stochastic FF patterns on the task (Figure 6C).

### 3.3 fMRI & PPI results

We next explored differences in BOLD amplitude and interference‐specific functional connectivity (PPI) in the two cohorts of ASD and TD participants that survived stringent matching on head movements and were also matched on the proportions of males and females of comparable ages.

ASD children from the ‘genetic’ sample showed more activation to I versus C trials (GLM1) (*p*_uncorr_ < .005, TFCE) in several areas, in particular in the cerebellum (MNI: 0, −49, −2; 6 voxels) and right Superior Parietal Lobule (SPL) (MNI: 21, −76, 58, 34 voxels) (Figure S2A). From the CEM sample, ASD individuals again showed increased activation to I trials versus C trials (*p*_uncorr_ < .005, TFCE) in several areas, including in the right lingual Gyrus (MNI: 12, −79, −11, 9 voxels), cerebellum (MNI: 0, −52, −35, 6 voxels), and right Inferior Temporal Gyrus (ITG, MNI: 54, −4, −35, 5 voxels) (Figure S2B). No other within‐ or between‐group level analyses survived FWE‐corrected (*p*_corr_ < .05) or uncorrected TFCE thresholding (*p*_uncorr_ < .001).

Our GLM2 model served as a check that all participants indeed showed activation to task alone. Here, within‐group analyses showed robust BOLD activation that survived stringent FWE‐corrected, TFCE threshold of 0.05 for ASD and TD groups in both cohorts (Figure S3A,B). However, no between‐group inferences were significant at the FWE‐corrected threshold (in the children's genetic cohort, TD group showed more activation relative to ASD at *p*_uncorr_ < .005, TFCE).

**Figure 7:**
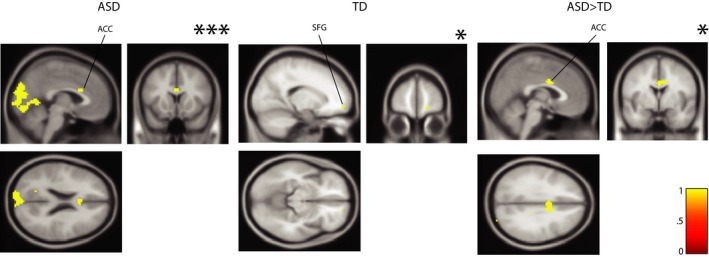
Permutation results from GLM3 in children‐only (‘genetic’) cohort assessing activation following (vs. preceding) decision on the task. Left panel shows maps from ASD children, middle panel shows maps from TD children, and right panel shows that ASD showed more activation relative to TD after making a decision on the task. Views shown are axial, sagittal, and coronal (clockwise). *indicates significance at *p *< .005; ***indicates significance at FWE‐corrected *p *< .001

Using GLM3, we assessed whether BOLD differs in ASD as a function of decision to respond (“phase 1” before the decision and “phase 2” after the decision). In the children's ‘genetic’ cohort, ASD children showed significant activation to phase 2 (vs. phase 1) in several areas, including Anterior Cingulate Cortex (ACC, MNI: −3, 14, 22, 17 voxels) and the cerebellum (FWE, *p*_corr_ < .001, TFCE). TD children showed activation in superior frontal gyrus (SFG) (MNI: 18, 56, −11, 5 voxels) and the right hippocampus (MNI: 39, −43, −5, 19 voxels) (*p*_uncorr_ < .005, TFCE). Notably, ASD children showed significantly more activation relative to TD children to phase 2 (vs. phase 1) in the ACC (MNI: 3, −1, 31; 45 voxels) (*p*_uncorr_ < .005, TFCE: Figures 7; S4A shows additional slices). Considering the CEM cohort, we found significant whole‐brain activation in ASD (FWE, *p*_corr_ < .0005, TFCE) and in TD (FWE, *p*_corr_ < .002, TFCE) (Figure S4B). However, between‐group differences for the CEM cohort did not survive thresholding (neither FWE‐corrected [*p*_corr_ < .05, TFCE] nor uncorrected TFCE [*p*_uncorr_ < .005] or [*p*_uncorr_ < .001]). (When considering contrast where activation was greater to phase 1 vs. phase 2 of the trial [phase 1 > phase 2], no within‐ or between‐group activation was detected in the ‘genetic’ children subgroup. Although within‐group activation was detected for both ASD [*p*_uncorr_ < .001, TFCE] and TD [*p*_uncorr_ < .001, TFCE] in the CEM subgroup, no between‐group analyses survived FWE‐corrected [*p*_corr_ < .05] or uncorrected TFCE thresholding [*p*_uncorr_ < .001]).

These results examining BOLD amplitude to task in ASD and TD were informative as follows. GLM2 showed robust within‐group BOLD amplitude to task in both cohorts, revealing whole‐brain activity during the entire trial, whereas GLM3 showed between‐group difference in activation during a postdecision stage in children with ASD. However, GLM1 did not reveal between‐group interference‐driven differences between ASD and TD. We next investigated interference‐specific functional coupling between key ROIs and the rest of the brain in ASD and TD controls in the two cohorts.

#### 3.3.1 Psychophysiological interaction

Examination of functional connectivity modulated by the interference condition (modulation of functional connectivity by task; I vs. C trials; PPI analysis) between 9 independently‐defined ROIs and the whole brain revealed significant between‐group differences in both ‘genetic’ and ‘cem’ cohorts. For all seeds probed, we found evidence for significantly greater within‐group (ASD, TD) temporal correlation to high‐interference, I, relative to low‐interference, C trials (*p*_uncorr_ < .005, TFCE), except for right AI‐FO seed in the ‘genetic’ cohort and for left IPS and left IFS seeds in the ‘cem’ ASD cohort which did not reach significance.

**Figure 8:**
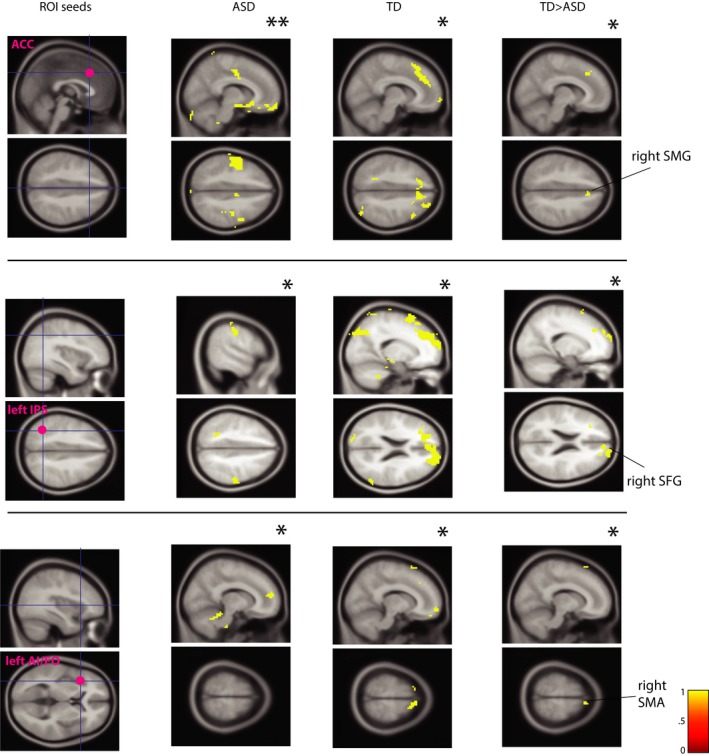
Permutation results for interference‐specific coupling (psychophysiological interaction, PPI analyses) for the children‐only ‘genetic’ imaging cohort (modulation of functional connectivity by task; I vs. C trials). Left‐most panel shows location of ROI seeds, followed by within‐ (ASD, TD) and between‐group (TD > ASD) PPI maps, all shown on MNI152 template. For each seed, sagittal (top) and axial (bottom) views are shown. Individuals with TD showed increased interference‐specific coupling relative to ASD participants. (Region of Interest (ROI) seeds, ACC: Anterior Cingulate Cortex; IPS: Inferior Parietal Sulcus; AI/FO: Anterior Insula/Frontal Operculum). *indicates significance at *p *< .005; **indicates significance at *p *< .001

We found that TD children in the ‘genetic’ cohort showed significantly greater interference‐sensitive coupling to high‐ relative to low‐interference trials than ASD children, in particular between the ACC and right Superior Medial Gyrus, SMG: MNI (9, 29, 40), 13 voxels [*p*_uncorr_ < .005, TFCE], between left IPS and the rest of the brain (including right Superior Frontal Gyrus, SFG: MNI [18, 68, 22], 319 voxels [*p*_uncorr_ < .005, TFCE]), as well as between left AI‐FO and right Supplementary Motor area ([MNI: 12, 29, 67], 6 voxels [*p*_uncorr_ < .005, TFCE]) (Figure 8).

In the ‘cem’ cohort, TD participants showed significantly greater coupling (relative to ASD participants) between the ACC and the rest of the brain (including left Middle Frontal Gyrus, MFG [MNI −39, 41, 22], 68 voxels and right thalamus [MNI 1, −16, 15], 42 voxels [*p*_uncorr_ < .005, TFCE]). Significantly greater coupling was found in TD between left IPS and the rest of the brain (left MFG [MNI −42, 44, 25], 17 voxels [*p*_uncorr_ < .001, TFCE]) (at *p*_uncorr_ < .005, TFCE, significantly greater between‐group co‐activation with ACC (MNI 0, 35, 31), 141 voxels and with right MFG (MNI 36, 62, 16), 185 voxels was also detected).

**Figure 9:**
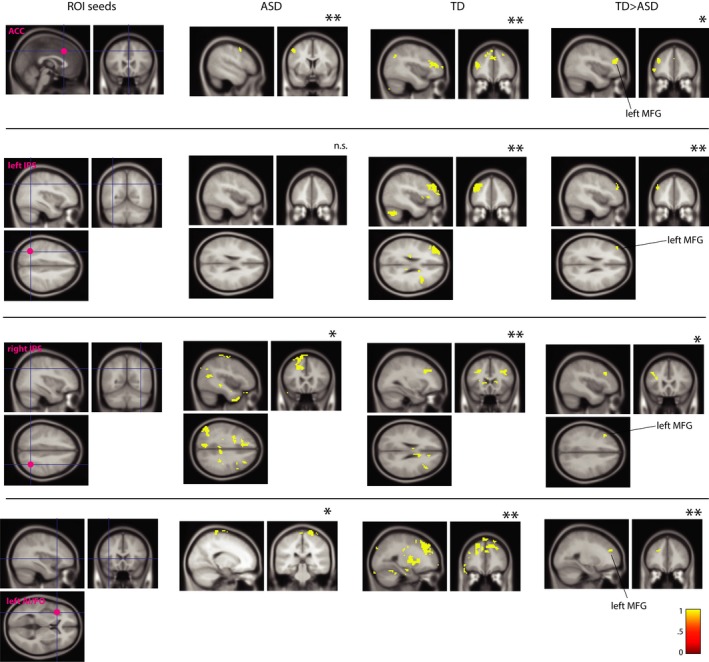
Permutation results for interference‐specific coupling (psychophysiological interaction, PPI analyses) for ‘cem’ imaging cohort. Left‐most panel shows location of ROI seeds, followed by within‐ (ASD, TD) and between‐group (TD > ASD) PPI maps, all shown on MNI152 template. For bilateral IPS seeds, axial, sagittal, and coronal (clockwise) views are shown. For ACC and left IA/FO seed, sagittal (left) and coronal (right) views are shown. Similar to children‐only cohort, individuals with TD in the ‘cem’ cohort showed increased interference‐specific coupling relative to ASD participants. (Region of Interest (ROI) seeds, ACC: Anterior Cingulate Cortex; IPS: Inferior Parietal Sulcus; AI/FO: Anterior Insula/Frontal Operculum). *indicates significance at *p *< .005; **indicates significance at *p *< .001

Significantly greater coupling was found in TD between right IPS seed and the rest of the brain (left MFG [MNI −30, 20, 16], 36 voxels, [*p*_uncorr_ < .005, TFCE]). We also detected significantly greater coupling in TD between left AI‐FO and the rest of the brain (including left MFG [MNI −24, 38, 25], 24 voxels [*p*_uncorr_ < .001, TFCE]) (significantly greater co‐activation with right MFG (MNI 27, 35, 25) was also detected at *p*_uncorr_ < .005, TFCE; 407 voxels) (Figure 9). No other seeds survived permutation testing at the between‐group level; there were no between‐group significant results where ASD>TD.

In summary, in the two cohorts stringently matched on the amount of movement during the scan, our permutation tests revealed significant between‐group difference in GLM3 in the ‘genetic’ cohort, where children with ASD showed significantly more activation in the ACC relative to TD children during postdecision task stage. Further, we found significant between‐group differences in interference‐specific temporal correlation between *a priori* defined ROI seeds and the rest of the brain. Permutation tests showed that 3 out of 9 PPI seeds for the ‘genetic’ cohort (ACC, left IPS, and left AI‐FO) and 4 out of 9 PPI seeds for the ‘cem’ cohort (ACC, left IPS, right IPS, and left AI‐FO) showed a between‐group difference, with significant regions including frontal areas (SMG, SFG, MFG), as well as subcortical areas. Thus, PPI analysis revealed significantly stronger modulation of functional coupling during high‐interference condition in the TD group compared to the ASD group, consistent for both ‘genetic’ and ‘cem’ cohorts.

Previous work implicates MFG (dlPFC) in top‐down signal biasing (Egner & Hirsch, 2005), mediation of sensory input and action (Suzuki & Gottlieb, 2013), and encoding uncertainty of action or choice selection (Rushworth & Behrens, 2008). Parietal cortex, specifically, the IPS (and its analog, lateral intraparietal area, LIP, in primates) supports modulation of attention (King, Korb, & Egner, 2012) and suppression of distracters (Gottlieb, 2007; Oristaglio, Schneider, Balan, & Gottlieb, 2006) as well as the “binding” or integration of visuospatial representations with task demands (Gottlieb, 2007; Oristaglio et al., 2006), reward expectations, and past experiences (Luo et al., 2010) to guide optimal response selection. ACC has been shown to be involved in involved in inhibitory control (Ridderinkhof, Nieuwenhuis, & Braver, 2007), motor planning (Nachev, Kennard, & Husain, 2008), action selection (Kuwabara, Mansouri, Buckley, & Tanaka, 2014) and Bayesian prediction errors in a stop‐signal task (Ide, Shenoy, Yu, & Li, 2013). Insula (e.g., Grinband, Hirsch, & Ferrera, 2006; Lamm & Singer, 2010) as well as IFS (e.g., Liu & Pleskac, 2011) have been implicated in decision making under uncertainty.

### 3.4 Linking behavioral uncertainty (trial‐to‐trial fluctuations) with interference‐specific neural coupling in CEM imaging cohort

**Figure 10:**
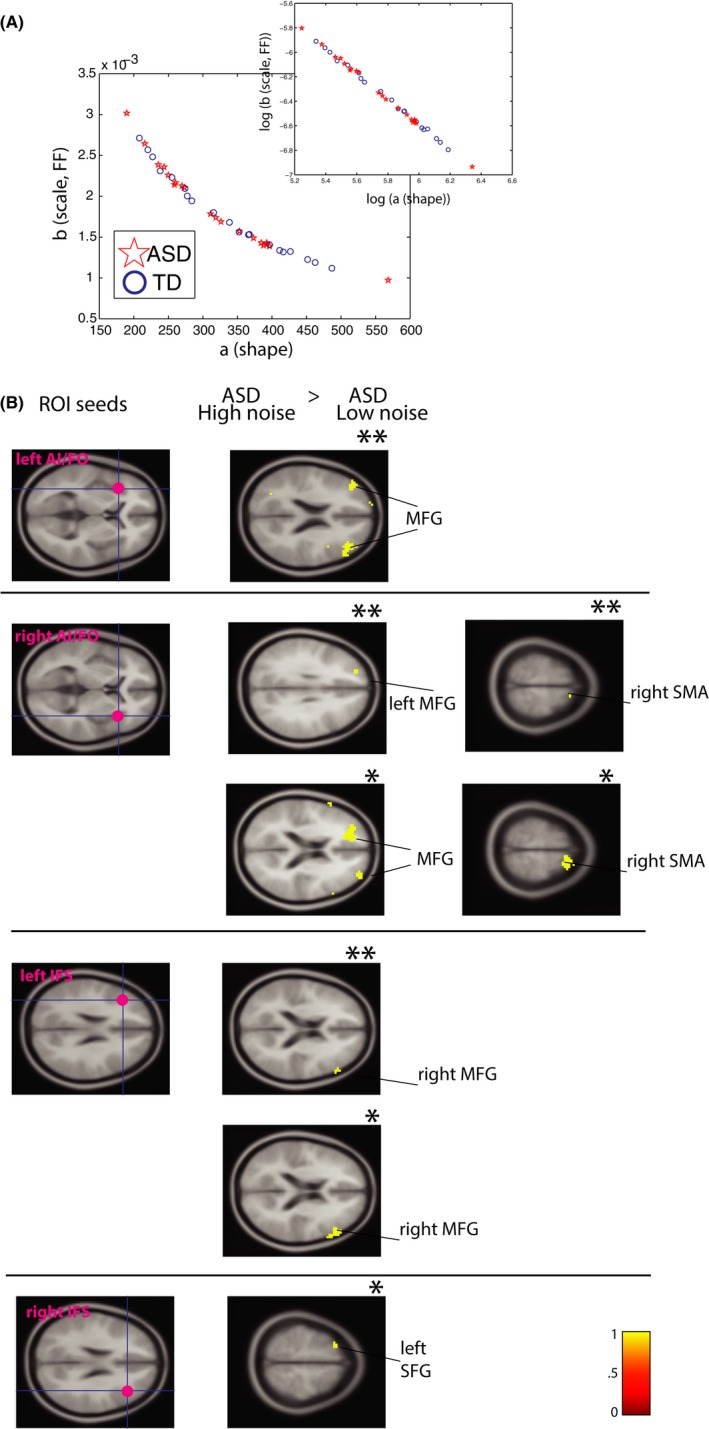
Brain‐behavior analyses in the ‘cem’ cohort. (A) Parameter estimates for trial‐to‐trial fluctuations on the Gamma parameter plane for 20 ASD and 20 TD (normalized peaks analysis) included in the imaging ‘cem’ cohort. (B). Permutation results for interference‐specific coupling (psychophysiological interaction, PPI analyses). Individuals with ASD with worse stochastic patterns (‘high noise’ subgroup) showed increased interference‐specific coupling between bilateral AI/FO and IFS seeds and prefrontal brain regions relative to individuals with ASD who showed more normative stochastic patterns during the scan. Note: ASD ‘high noise’ subgroup is comprised of those ASD individuals shown in (A) with high *b* and low *a* parameter estimates (leftward shift on the x‐axis and upward shift on the y‐axis) whereas ASD ‘low noise’ subgroup is comprised of those with low *b* and high *a* parameter estimates (rightward shift on the x‐axis, downward shirt on the y‐axis). (Region of Interest (ROI) seeds, AI/FO: Anterior Insula/Frontal Operculum; IFS: Inferior Frontal Sulcus). *indicates significance at *p *< .005; **indicates significance at *p *< .001

We next examined the role of individual differences in the statistical nature of stochastic patterns in behavior, during the scan, in interference‐specific neural coupling between ROI seeds and the rest of the brain in our largest (‘cem’) imaging cohort. Although individuals with ASD were carefully and nonparametrically matched to TD healthy controls on age, sex, and amount of movement during the scan, we nevertheless wanted to investigate whether individuals with ASD who showed differing amounts of noise‐to‐signal levels (uncertainty) in their behavioral response fluctuations during the fMRI scan may differ at the neural level (i.e., in how they instantiate task demands during the scan) (Figure 10A shows that both ASD and TD in this cohort had overlapping (no between‐group significance) parameter estimates on the Gamma parameter plane of trial‐to‐trial behavioral fluctuations; we obtained similar subgroupings when splitting on median score using either the *a* or *b* parameter).

When examining interference‐specific coupling in individuals regardless of diagnostic status, no difference was found, using any of seeds, between subgroups that were especially high on behavioral noise‐to‐signal levels versus those with lower levels. However, examining participants by diagnostic status, ASD versus TD, we found that the influence of behavioral variability levels during the task was differently reflected in neural temporal correlation between seeds and the rest of the brain for ASD and TD individuals. Specifically, we did not detect a difference in interference‐specific coupling, using any of the seeds, between TD individuals who were high versus low in their noise levels during the task.

In contrast, individuals with ASD with higher relative to lower levels of noise (high > low) showed significantly greater interference‐specific coupling between bilateral AI‐FO and bilateral IFS seeds and the rest of the brain (Figure 10B). Specifically, we found greater coupling between left AI‐FO and areas including bilateral MFG (right MFG: MNI 51, 32, 19, 184 voxels and left MFG: MNI −39, 38, 22, 67 voxels) (*p*_uncorr_ < .001, TFCE; 1 right IPL cluster also significant at FWE, *p*_corr_ < .05, TFCE). We found significantly greater coupling between right AI‐FO and left MFG (MNI −18, 44, 19, 29 voxels) and right SMA (MNI 13, 16, 67, 6 voxels) (*p*_uncorr_ < .001, TFCE). In addition, we found significantly greater coupling between left IFS and right MFG (MNI 57, 32, 19, 9 voxels, *p*_uncorr_ < .001, TFCE), and between right IFS and left SFG (MNI −24, 23, 61, 11 voxels, *p*_uncorr_ < .005, TFCE). No other seeds reached significance where high > low noise ASD subgroups; there were no significant seeds where low > high. Thus, those ASD individuals with worse stochastic fluctuation patterns (relative to those ASD individuals with more normative statistical features) showed significantly *increased* interference‐specific coupling between bilateral AI‐FO and bilateral IFS seeds and the rest of the brain, in particular, bilateral MFG.

## 4 Discussion

We used functional MRI, a seed‐based analysis of neural coupling, and stochastic analyses of subtle behavioral manifestations to investigate the coordination of top‐down and feed‐forward processing in ASD. We found evidence for a noisier and more random response structure and higher noise‐to‐signal levels (worse Fano Factor, FF) in empirically estimated distributional parameters characterizing stochastic signatures in fluctuations of responses in ASD (Figure 5A), which were worse in ASD adults with higher ADOS clinical scores (Figure 6A). In the 2 stringently matched imaging cohorts (matched on amount of movement during the scan, sex, and age), our permutation analyses revealed significant between‐group differences in interference‐driven functional connectivity between ASD and TD. While ASD participants showed significantly weaker interference‐specific functional connectivity between several independently defined ROI seeds, including ACC and IPS and the rest of the brain, when processing trials with a high versus low level of interference relative to TD individuals (Figures 8 and 9), significant individual differences emerged when examining patterns of behavioral fluctuations during the task in ASD individuals. ASD individuals who showed higher uncertainty on the task showed *increased* interference‐specific coupling between bilateral anterior insula and IFS seeds and prefrontal cortex (Figure 10B).

Empirically estimated parameters of subtle fluctuations (i.e., trial‐to‐trial variability) in latencies in ASD were non‐Gaussian on the Gamma parameter plane (i.e., shifted toward the more random, Exponential distribution), and thus the stochastic structure of fluctuations is noisier and unpredictable relative to the more normative shape of TD distribution. Specifically, individuals with ASD showed a failure to apply or incorporate information from prior trials on the Simon task, across both stimulus types and therefore across both levels of cognitive interference, to provide a normative response. In contrast to TD individuals whose response characteristics tended toward the more normal Gaussian distribution, patterns of ASD individuals revealed statistical features that tended toward a more Exponential, “memoryless” distribution; Figure 5A. Unlike previous studies proposing higher variability in neural responses and sensory processing in ASD (e.g., Dinstein et al., 2012; Haigh, Minshew, Heeger, Dinstein, & Behrmann, 2016; Torres et al., 2016; Weinger, Zemon, Soorya, & Gordon, 2014), we discovered evidence for worse noise‐to‐signal at two distinct levels of functioning in adults with ASD, linking worse spontaneous movement‐based FF during the scan and worse response fluctuation‐based FF in RT latencies at the same time (Figure 6C). Finally, in our CEM imaging cohort, we linked differences in stochastic patterns in behavior during the scan to significant differences in interference‐specific neural coupling between bilateral anterior insula and IFS and prefrontal cortex in ASD (Figure 10B).

Fluctuation in degree of reliability or confidence (i.e., “precision”; Lawson, Friston, & Rees, 2015; Lawson, Rees, & Friston, 2014) at lower levels relative to top‐down, prior inferences or beliefs may lead to a difficulty in contextualizing available evidence at lower levels of the cortical hierarchy in ASD (Lawson et al., 2014). For example, noisier signatures of afferent somatosensory signals from the button‐press arriving to the brain (via the brainstem or the spinal cord) may make it more difficult to determine the weight to be placed on inputs arriving via photoreceptors (i.e., considered in relation to higher‐level, top‐down goals). In this sense our findings of increased (internal) noise levels, in all participants with ASD, may contribute to an aberrant precision‐weighting of available evidence relative to top‐down inference dictated by higher‐level task demands. Under this possibility, atypically weighted internal, and not necessarily environmental, uncertainty poses a risk for fidelity of information transfer across the CNS in autistic individuals.

Examining differences in BOLD amplitude before and after a response decision, we found evidence for a protracted BOLD in the ACC in children with ASD relative to TD children. One possibility is that processing of information in children with ASD is characterized by greater dispersion, or alternatively, by continuing to think about the task even after the decision has already been made. In children with ASD, the inability to contextualize flexibly (i.e., to suppress or attenuate) competition among the visual and spatial dimensions of the same stimulus within lower‐level parietal cortices, despite the task‐irrelevance of one of the dimensions, may have required compensatory increases in activation at higher levels of the cortical hierarchy, in the ACC (i.e., via the gain or sensitivity of postsynaptic responses; Lawson et al., 2014) (Figure 7; this finding is considered preliminary given the small size of this sample).

Functional connectivity was atypically modulated by increased level of cognitive interference during the scan in ASD relative to TD individuals (findings consistent in the two “homogenized” cohorts whose data could be considered reliable for neuroimaging analyses). We found significantly reduced interference‐specific modulation of connectivity in ASD relative to TD participants between the seeds of ACC, left IPS, and left AI‐FO and brain regions including right SMG and right SFG in the children‐only cohort, and reduced connectivity between ACC, left and right IPS, and left AI/FO and regions including left MFG and right thalamus in the ‘cem’ cohort. We consider this result as follows. First, it speaks to our hypotheses that individuals with ASD exhibit atypical communication of task demands across the brain. In other words, in ASD, the levels of “connectivity” between cortical circuits do not seem to be sensitive to differences in information processing demands in a similar way as for TD, when autistic individuals perform an interference task with well‐defined rules. In this regard, these findings are consistent with theories of aberrant neural connectivity in ASD (Belmonte et al., 2004; Brock et al., 2002; Just, Keller, Malave, Kana, & Varma, 2012; Just et al., 2004). In a different sense, it may be that the fMRI time courses in ASD have higher noise‐to‐signal levels (i.e., high variance relative to BOLD amplitude) which could produce a weaker representation of differences (hence, weaker modulation) between the different task conditions. (Note that although ASD individuals could perform the task and distinguish between I and C trials (Figure 3), their performance in general relative to TD participants was noisier and more random).

We further considered the possibility that as a group, ASD individuals were more heterogeneous, with distinct individual‐specific temporal coupling signatures, whereas individuals with TD would show overall more similar temporal coupling signatures. Analysis of the role of levels of noise in stochastic behavioral patterns (i.e., ‘high’ vs. ‘low’ subgroups in the CEM imaging cohort, which had enough participants to permit brain‐behavior analyses) showed no effect of increased noise levels in behavior on interference‐specific neural coupling in TD participants, but a significant difference was discovered in ASD. Individuals with ASD with worse stochastic patterns in behavior during the scan showed significantly *increased* interference‐specific coupling between bilateral AI‐FO and IFS seeds and the rest of the brain (Figure 10B). These findings suggest that in ASD, individual differences in experiencing subtle (internal) uncertainty are linked to atypical neural processing of stimulus‐driven (external or environmental) uncertainty, in particular between bilateral anterior insula and MFG.

Because the brain constructs a representation of the physical world for adaptive perception and action by detecting biologically useful (both spatial and temporal) regularities in the environment, for example, in the context of visual perception (e.g., Denisova, Feldman, Su, & Singh, 2016) and continuously updates and integrates sensory inputs arriving to CNS via a feedback loop, both external and internal uncertainty may affect the integrity of this process. The temporal synchronization of brain activity may be especially helpful under more complex, underdetermined (Feldman, 2013), and uncertain task conditions, as when processing high‐interference trials in the current study, because evaluating sensory evidence and ascertaining proper action in such conditions is likely to entrain remote neuronal assemblies, including those in higher‐order brain areas such as the prefrontal cortex (Huettel, Song, & McCarthy, 2005). Atypical interference‐specific co‐activation during routine information processing in ASD may affect the functional integration and transfer of information that is needed for the resolution of feed‐forward and top‐down information processing demands in support of adaptive actions, and may help explain, in part, why individuals with ASD often have difficulty with resolving these competing demands when transitioning between activities (Forest, Horner, Lewis‐Palmer, & Todd, 2004; Schreibman, Whalen, & Stahmer, 2000), regulating emotions, and containing verbal and physical aggression (Farmer & Aman, 2011; Loveland & Tunali‐Kotoski, 2005; Mazefsky et al., 2013). More generally, atypical interference‐specific modulation of functional connectivity may impair the ability of ASD individuals to form an optimal representation of their world and to resolve or minimize computational costs of negotiating competing internal and external demands.

Our findings should be considered within the context of evidence from numerous genetic and neurobiological studies suggesting the presence of synaptic and axonal pathology in ASD (Bourgeron, 2009; McFadden & Minshew, 2013; Zoghbi, 2003). For example, known genetic mutations that produce ASD predominantly affect the development or function of synapses (e.g., Betancur, 2011; Jamain et al., 2003; Zoghbi & Bear, 2012). Prior postmortem studies have reported exuberant cellular and synaptic growth and increased number of neurons in the PFC (Courchesne et al., 2011), aberrant synaptic density and connectivity (Fatemi, Reutiman, Folsom, & Thuras, 2009; Hutsler & Zhang, 2010), synaptic pruning deficits (Tang et al., 2014), and reduced numbers of long‐range, large axons together with increased numbers of thin white matter axons (Zikopoulos & Barbas, 2010) in the brains of persons with ASD.

Including ASD participants with IQ > 70 may have limited somewhat the generalizability of our findings. However, others have noted that autistic individuals, even those with similar cognitive abilities, are generally less well functioning in their daily routines than TD individuals (Pellicano & Stears, 2011). Here we found significant between‐group differences in the stochastic patterns of subtle fluctuations and interference‐specific neural coupling, even in individuals with ASD who have normal IQ.

## 5 Conclusion

Subtle and informative differences in the structure of experiencing information exist between individuals with ASD and TD controls across multiple levels of inquiry. Stochastic patterns in task‐driven behavior were worse in individuals with ASD relative to TD participants, in particular in adults with ASD who had higher clinical scores as well as, notably, in those who had worse noise‐to‐signal levels in spontaneous movements during the scan. In the two age and sex‐matched imaging cohorts that were also stringently matched on the amount of movement during the scan (using objective criteria: excluding datasets from participants with movement spikes over 1 mm or 1^o^, and requiring a 0.2 mm cut‐off on mean FD per participant, resulting with <0.002 mm between‐group difference on FD in both imaging cohorts; *p *> .9), functional connectivity patterns ASD were atypically modulated by the level of interference during the scan relative to TD individuals, revealing a less parsimonious, atypical communication of task‐driven demands across the brain. When considering all ASD individuals regardless of the statistical nature of their behavioral stochastic patterns, we found an overall weaker interference‐specific modulation relative to TD individuals, in particular between the seeds of ACC and IPS and the rest of the brain. However, relative to those individuals with ASD who showed more normative stochastic patterns in behavior during the scan, individuals with ASD with higher noise‐to‐signal levels showed *increased* interference‐specific coupling, in particular, between bilateral anterior insula and prefrontal cortex. This finding suggests a (functional) neural link when experiencing heightened levels of uncertainty in individuals with ASD. In atypical neurodevelopment in humans, internal and external uncertainty is more tightly linked than previously thought.

## Conflict of Interest

The authors declare no competing financial interests. Specifically, all authors report that they have no conflicts of interest, including any financial interests, activities, relationships and affiliations that could have influenced the authors’ decisions, work, or manuscript: Dr. Denisova, Mr. Zhao, Dr. Wang, Dr. Goh, Mr. Huo, and Dr. Peterson report no competing interests.
