# Supplementary material for: Cortical interactions during the resolution of information processing demands in autism spectrum disorders
Source: Brain Behav. 2016 Dec 24;7(2):e00596. doi: 10.1002/brb3.596 (PMC5318360; doi:10.1002/brb3.596)
Supplement: Supplementary file 5 [file BRB3-7-e00596-s005.docx]

**S1 Table.**

| groupID | examID | age | sex | Mean_FDmotion | Mean_Rel_RMS |
| --- | --- | --- | --- | --- | --- |
| 1 | 1615 | 20.21 | 0 | 0.1147 | 0.0591 |
| 1 | 4825 | 20.59 | 1 | 0.1906 | 0.0913 |
| 1 | 3594 | 17.92 | 0 | 0.1374 | 0.0722 |
| 1 | 6242 | 19.00 | 0 | 0.1529 | 0.0780 |
| 1 | 3498 | 16.34 | 0 | 0.0959 | 0.0505 |
| 1 | 6707 | 18.71 | 0 | 0.0801 | 0.0419 |
| 1 | 4286 | 14.64 | 0 | 0.1409 | 0.0761 |
| 1 | 4754 | 14.89 | 0 | 0.0735 | 0.0386 |
| 1 | 3942 | 12.47 | 0 | 0.1628 | 0.0860 |
| 1 | 3306 | 11.41 | 0 | 0.2069 | 0.1083 |
| 1 | 3823 | 10.88 | 0 | 0.1567 | 0.0830 |
| 1 | 4910 | 11.50 | 1 | 0.1208 | 0.0618 |
| 0 | 5250 | 10.40 | 1 | 0.1802 | 0.0813 |
| 0 | 5877 | 20.33 | 0 | 0.1129 | 0.0569 |
| 0 | 5705 | 19.24 | 1 | 0.1360 | 0.0720 |
| 0 | 5974 | 18.65 | 0 | 0.1766 | 0.0842 |
| 0 | 5507 | 18.27 | 0 | 0.1363 | 0.0808 |
| 0 | 4063 | 13.36 | 0 | 0.0724 | 0.0366 |
| 0 | 6399 | 14.93 | 0 | 0.1212 | 0.0636 |
| 0 | 6268 | 12.60 | 0 | 0.1597 | 0.0884 |
