# Supplementary material for: Cortical interactions during the resolution of information processing demands in autism spectrum disorders
Source: Brain Behav. 2016 Dec 24;7(2):e00596. doi: 10.1002/brb3.596 (PMC5318360; doi:10.1002/brb3.596)
Supplement: Supplementary file 6 [file BRB3-7-e00596-s006.docx]

**S2 Table.**

| groupID | examID | age | sex | Mean_FDmotion | Mean_Rel_RMS |
| --- | --- | --- | --- | --- | --- |
| 1 | 3929 | 45.82 | 0 | 0.1647 | 0.0972 |
| 1 | 5493 | 39.49 | 0 | 0.1285 | 0.0678 |
| 1 | 2041 | 28.82 | 0 | 0.1278 | 0.0682 |
| 1 | 6032 | 27.99 | 1 | 0.1703 | 0.0784 |
| 1 | 6176 | 27.76 | 0 | 0.0872 | 0.0471 |
| 1 | 6353 | 26.69 | 0 | 0.0869 | 0.0479 |
| 1 | 2626 | 22.34 | 0 | 0.0967 | 0.0494 |
| 1 | 1615 | 20.21 | 0 | 0.1147 | 0.0591 |
| 1 | 5570 | 23.87 | 0 | 0.0532 | 0.0292 |
| 1 | 4925 | 22.15 | 0 | 0.1792 | 0.0931 |
| 1 | 6205 | 22.98 | 0 | 0.1435 | 0.0747 |
| 1 | 5954 | 21.43 | 0 | 0.0946 | 0.0522 |
| 1 | 3594 | 17.92 | 0 | 0.1374 | 0.0722 |
| 1 | 6242 | 19.00 | 0 | 0.1529 | 0.0780 |
| 1 | 3498 | 16.34 | 0 | 0.0959 | 0.0505 |
| 1 | 6707 | 18.71 | 0 | 0.0801 | 0.0419 |
| 1 | 4286 | 14.64 | 0 | 0.1409 | 0.0761 |
| 1 | 4754 | 14.89 | 0 | 0.0735 | 0.0386 |
| 1 | 3942 | 12.47 | 0 | 0.1628 | 0.0860 |
| 0 | 4406 | 42.99 | 0 | 0.1791 | 0.1010 |
| 0 | 2146 | 40.30 | 0 | 0.1401 | 0.0741 |
| 0 | 2575 | 31.08 | 0 | 0.1328 | 0.0669 |
| 0 | 5630 | 28.63 | 0 | 0.0841 | 0.0402 |
| 0 | 6170 | 27.57 | 1 | 0.1654 | 0.0912 |
| 0 | 6087 | 25.72 | 0 | 0.0714 | 0.0364 |
| 0 | 5637 | 25.30 | 0 | 0.1317 | 0.0718 |
| 0 | 5586 | 24.24 | 0 | 0.0583 | 0.0300 |
| 0 | 5422 | 23.55 | 0 | 0.1211 | 0.0628 |
| 0 | 5682 | 23.55 | 0 | 0.1089 | 0.0557 |
| 0 | 6156 | 22.44 | 0 | 0.1165 | 0.0616 |
| 0 | 5946 | 21.63 | 0 | 0.0790 | 0.0395 |
| 0 | 5877 | 20.33 | 0 | 0.1129 | 0.0569 |
| 0 | 2502 | 16.30 | 0 | 0.1639 | 0.0805 |
| 0 | 5974 | 18.65 | 0 | 0.1766 | 0.0842 |
| 0 | 5507 | 18.27 | 0 | 0.1363 | 0.0808 |
| 0 | 4063 | 13.36 | 0 | 0.0724 | 0.0366 |
| 0 | 6399 | 14.93 | 0 | 0.1212 | 0.0636 |
| 0 | 4195 | 12.70 | 0 | 0.1399 | 0.0701 |
| 0 | 6268 | 12.60 | 0 | 0.1597 | 0.0884 |
