# Supplementary material for: Cortical interactions during the resolution of information processing demands in autism spectrum disorders
Source: Brain Behav. 2016 Dec 24;7(2):e00596. doi: 10.1002/brb3.596 (PMC5318360; doi:10.1002/brb3.596)
Supplement: Supplementary file 7 [file BRB3-7-e00596-s007.docx]

**Supplemental Materials**

**Captions to Supplementary Figures and Tables**

**Captions to Supplementary Figures**

**Supplemental Figure 1.** Higher noise-to-signal levels of spontaneous movement during the scan. **(A).** Empirical Cumulative Distribution Functions (eCDFs) of ASD and TD distributions were significantly different (Kolmogorov-Smirnov test p=1.4075e-100) (panels show data on each frame for ASD (left; 20,137 frames) and TD (right; 20,414 frames) participants. **(B).** Parameter estimates on the Gamma plane for ASD and TD movement distributions (non-overlapping 95% CIs for both shape and scale parameters). **(C).** At the individual participants’ level, ASD participants have significantly higher noise-to-signal levels (scale parameter) relative to TD individuals.

**Supplemental Figure 2.** Permutation results from GLM1 (“I vs. C” model, “I>C” contrast) for 2 imaging cohorts. **(A)**. ‘genetic’ (children’s cohort), ASD within-group (p_uncorr_<0.001, TFCE) **(B).** ‘CEM’ (children, adolescents, and adults cohort), ASD within-group (p_uncorr_<0.005, TFCE). No other within- or between-group level analyses survived FWE-corrected (p_corr_<0.05) or uncorrected TFCE thresholding (p_uncorr_<0.001).

**Supplemental Figure 3.** Permutation results from GLM2 (“full trial” model) for 2 imaging cohorts. **(A)** shows ‘genetic’ (children’s cohort) and **(B)** shows ‘CEM’ (children, adolescents, and adults cohort). FWE-corrected, TFCE threshold of p<0.05 for left and middle panels (within-group maps). No between-group inferences (right panel) were significant at the FWE-corrected threshold (one exception was in the children’s genetic cohort in A: TD group showed more activation relative to ASD at p_uncorr_<0.005, TFCE).

**Supplemental Figure 4.** Permutation results from GLM3 (“phase1 vs. phase2” model; “phase2>phase1” contrast) for 2 imaging cohorts. **(A)** shows ‘genetic’ (children’s) cohort. Left panel shows maps from ASD children (FWE, p_corr_<0.001, TFCE), middle panel shows maps from TD children (p_uncorr_<0.005, TFCE), and right panel shows that ASD showed more activation relative to TD (p_uncorr_<0.005, TFCE). **(B)** shows permutation results from the ‘CEM’ (children, adolescents, and adults) cohort. Left panel shows maps from ASD CEM cohort (FWE, p_corr_<0.0005, TFCE); middle panel shows maps from TD CEM cohort (FWE, p_corr_<0.002, TFCE). Between-group differences for the CEM cohort did not survive thresholding (neither FWE-corrected (p_corr_<0.05) nor uncorrected TFCE thresholding (p_uncorr_<0.001) or (p_uncorr_<0.001)).

**Captions to Supplementary Tables**

**Supplementary Table 1.** Matched values for ‘genetic’ children’s cohort used in the neuroimaging analyses. Please note: listed RMS values are presented, but were not used for matching. Group 1 indicates ASD, Group 0 indicates TD.

**Supplementary Table 2.** Matched values for ‘coarse exact match’ cohort used in the neuroimaging analyses. Please note: listed RMS values are presented, but were not used for matching. Group 1 indicates ASD, Group 0 indicates TD.
